# A long isoform of GIV/Girdin contains a PDZ-binding module that regulates localization and G-protein binding

**DOI:** 10.1016/j.jbc.2021.100493

**Published:** 2021-03-03

**Authors:** Jason Ear, Amer Ali Abd El-Hafeez, Suchismita Roy, Tony Ngo, Navin Rajapakse, Julie Choi, Soni Khandelwal, Majid Ghassemian, Luke McCaffrey, Irina Kufareva, Debashis Sahoo, Pradipta Ghosh

**Affiliations:** 1 Department of Cellular and Molecular Medicine, University of California San Diego, La Jolla, California, USA; 2 Biological Sciences Department, California State Polytechnic University, Pomona, California, USA; 3 Pharmacology and Experimental Oncology Unit, Cancer Biology Department, National Cancer Institute, Cairo University, Cairo, Egypt; 4 Skaggs School of Pharmacy and Pharmaceutical Sciences, University of California San Diego, La Jolla, California, USA; 5 Department of Pediatrics, University of California San Diego, La Jolla, California, USA; 6 Department of Chemistry and Biochemistry, University of California San Diego, La Jolla, California, USA; 7 Rosalind and Morris Goodman Cancer Research Centre, McGill University, Montreal, Canada; 8 Gerald Bronfman Department of Oncology, McGill University, Montreal, Canada; 9 Department of Medicine, University of California San Diego, La Jolla, California, USA; 10 Rebecca and John Moore Comprehensive Cancer Center, University of California San Diego, La Jolla, California, USA; 11 Veterans Affairs Medical Center, La Jolla, California, USA

**Keywords:** cell junctions, cell signaling, protein interactions, cancer, cell biology, GEM, guanine-nucleotide exchange modulator, PBM, PDZ-binding motif, PNS, post nuclear supernatant, UPLC, ultrahigh-pressure liquid chromatography

## Abstract

PDZ domains are one of the most abundant protein domains in eukaryotes and are frequently found on junction-localized scaffold proteins. Various signaling molecules bind to PDZ proteins *via* PDZ-binding motifs (PBM) and fine-tune cellular signaling. However, how such interaction affects protein function is difficult to predict and must be solved empirically. Here we describe a long isoform of the guanine nucleotide exchange factor GIV/Girdin (CCDC88A) that we named *GIV-L*, which is conserved throughout evolution, from invertebrates to vertebrates, and contains a PBM. Unlike GIV, which lacks PBM and is cytosolic, GIV-L localizes onto cell junctions and has a PDZ interactome (as shown through annotating Human Cell Map and BioID-proximity labeling studies), which impacts GIV-L's ability to bind and activate trimeric G-protein, Gαi, through its guanine-nucleotide exchange modulator (GEM) module. This GEM module is found exclusively in vertebrates. We propose that the two functional modules in GIV may have evolved sequentially: the ability to bind PDZ proteins *via* the PBM evolved earlier in invertebrates, whereas G-protein binding and activation may have evolved later only among vertebrates. Phenotypic studies in Caco-2 cells revealed that GIV and GIV-L may have antagonistic effects on cell growth, proliferation (cell cycle), and survival. Immunohistochemical analysis in human colon tissues showed that GIV expression increases with a concomitant decrease in GIV-L during cancer initiation. Taken together, these findings reveal how regulation in GIV/CCDC88A transcript helps to achieve protein modularity, which allows the protein to play opposing roles either as a tumor suppressor (GIV-L) or as an oncogene (GIV).

Scaffolding proteins are important molecules that regulate the temporal, spatial, and kinetic aspects of protein complex assembly (1, 2). Their multimodular makeup is key to regulating their local protein concentrations, proximity to, and subcellular dispositions (3). These functions of scaffold proteins allow for the biochemical properties of the target proteins to impart intracellular signaling plasticity in a dynamic and spatially restricted manner—earning scaffold proteins the reputation of “placemakers” and “pacemakers” of cell signaling (4). Among the numerous modules that facilitate scaffolding, PDZ domains (Postsynaptic density protein [PSD95], Drosophila disc large tumor suppressor [Dlg1], and Zonula occludens-1 protein [zo-1]) comprise one of the largest and are frequently encountered in proteins on cell–cell junctions (5). PDZ-binding motifs (PBMs) are short linear motifs commonly found on the C terminus of proteins (although internal PBMs do exist) and mediate the PDZ·PBM interactions.

Members of the CCDC88 family of proteins are multimodular molecular scaffolds that serve as signal transducers in eukaryotes (6, 7). In mammals, this family is comprised of three members: CCDC88A/GIV, CCDC88B/GIPIE, and CCDC88C/Daple; each member features a conserved HOOK-like domain and a coiled-coil domain on their N-terminal end; however, when it comes to the C-terminal end, there is more sequence divergence. In mammals, both GIV and Daple have a disordered C-terminal region, which contain a G-protein exchange and modulator (GEM) motif that they use to bind and activate the heterotrimeric G-proteins of the Gi subfamily (8). GIPIE, on the other hand, has a shortened C-termini and lacks the GEM motif. Daple is unique from GIV in that it contains a PBM, allowing it to bind to disheveled (Dvl), a key regulator in Wnt signaling. Furthermore, Daple contains a Frizzled-binding module, which enables binding to Wnt receptors (Frizzled/Fzd) (9, 10); in doing so, Daple links G-protein signaling to Wnt/Fzd signaling pathways (6, 7, 8, 9). The PBM module on Daple is also required for its localization to cell–cell junctions, *via* its ability to bind PDZ domain containing junctional proteins (PARD3, mPDZ, etc.), and such localization appears to be regulated by protein phosphorylation (11, 12).

Like Daple, GIV has also been observed on cell–cell junctions and has been found to interact with junction-associated polarity proteins in mammalian cells (13, 14, 15, 16). How it does so is unclear, especially because GIV in vertebrates was never reported to have the PBM module (6, 7), yet it is this same module on Daple that is responsible for its localization onto cell–cell junctions (11, 12). Interestingly, in invertebrates such as *C. elegans* and *Drosophila* (which lack Daple), GIV lacks its GEM motif, but contains a PBM, which has been shown to regulate cilia function in *C. elegans* (14, 17, 18).

Here we report the discovery of a novel isoform of GIV in vertebrates that contains both a G-protein modulatory, *i.e.*, the “GEM” motif, and a conserved C-terminal PBM. This isoform not only offers insights into the evolution of the gene between invertebrates and vertebrates, but also sheds light onto a mechanism that helps enrich GIV onto cell–cell junctions. Finally, through meta-analysis of publicly available interaction data and through GIV biotin proximity labeling (BioID), we identified a GIV-“PDZome” interaction network. We propose that the compartmentalization of the two GIV isoforms may explain the dual role of GIV as both a tumor suppressor and an oncogenic driver, as previously reported in the literature (19).

## Results

### GIV has a PDZ-binding motif that is evolutionarily conserved among vertebrates and invertebrates

**Figure 1:**
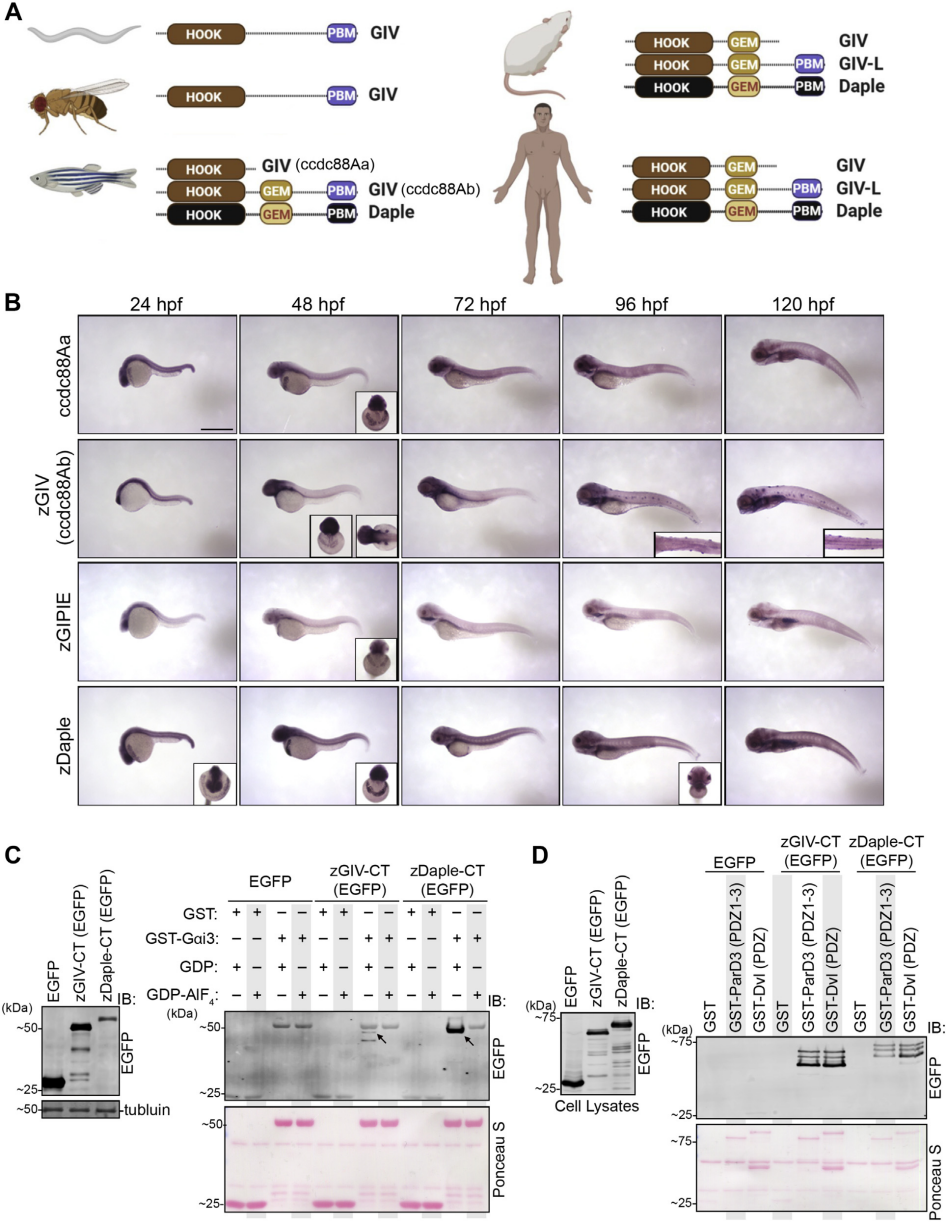
**The C terminus of GIV has an evolutionarily conserved functional PDZ-binding motif downstream of its G-protein binding and/or modulatory domains.***A*, schematic depicting the major modules and motifs within GIV and Daple across different species. *B*, whole-mount RNA *in situ* hybridization of the CCDC88 gene family in developing zebrafish embryos across multiple time points. Inset shows anterior or dorsal view of select embryos. Scale bar = 1 mm. *C*, GST-pull-down assays were carried out using purified rat Gαi3 (loaded with GDP or GDP-AlF_4_^−^) and lysates of HEK293T cells exogenous expressing zebrafish GIV-CT or Daple-CT. Bound proteins were analyzed (*right*) and equal loading of lysates was confirmed (*left*) by immunoblotting (IB). *Arrows* point to EGFP-zGIV-CT or EGFP-Daple-CT. *D*, GST-pull-down assays were carried out using purified GST-tagged PDZ domains of ParD3 and Dvl and lysates of HEK293T cells exogenous expressing zebrafish GIV-CT or Daple-CT and bound proteins were visualized as in *C*. GEM, guanine nucleotide-exchange modulator; GIV-L, long isoform of GIV; HOOK, a highly conserved microtubule-binding N-terminal domain; PBM, PDZ-binding motif.

**Figure 3:**
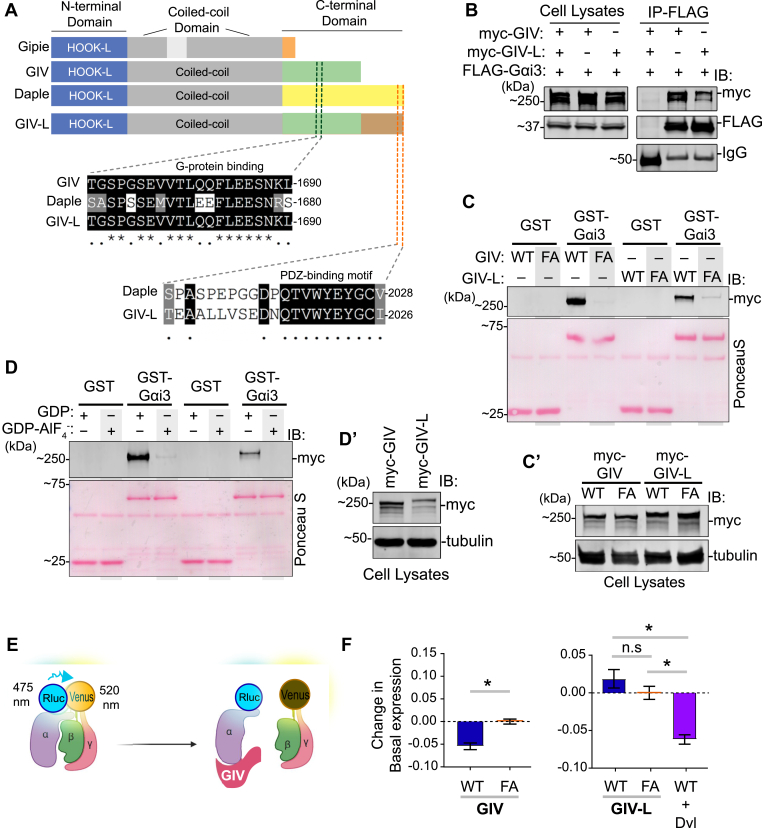
**Both GIV and GIV-L use their GEM motifs to preferentially bind GDP-bound Gαi, but only GIV WT nor other GIV variants, to reduce basal Gαi-RLuc2/mVenus-Gbg BRET in HEK293T cells.***A*, a schematic displaying the modular makeup of the CCDC88 family of proteins, from *top* to *bottom*—CCDC88A/GIV, CCDC88B/Gipie, and CCDC88C/Daple. *B*, equal aliquots of lysates of HEK293T cells coexpressing FLAG-tagged Gαi3 and either myc-tagged GIV or GIV-L constructs were subjected to immunoprecipitation assays using an anti-FLAG antibody. Bound proteins and cell lysates were assessed for Gαi3 (FLAG) and GIV (myc) by immunoblotting (IB). *C*, GST-pull-down assays were carried out using purified GST-Gαi3 and lysates of HEK293T cells exogenous expressing myc-tagged wild-type (WT) or F1685A mutant (FA) of human GIV or GIV-L. Bound GIV was analyzed by immunoblotting (IB) using an anti-myc antibody. *Panel C′* shows expression of proteins in the HEK293T cell lysates that were used as source of GIV in pull-down assays. *D*, GST-pull-down assays were carried out using purified GST-Gαi3, preloaded with GDP or GDP-AlF_4_^−^, and lysates of HEK293T cells exogenously expressing myc-tagged wild-type human GIV or GIV-L. *Panel D’*′shows expression of proteins in the HEK293T cell lysates that were used as source of GIV in pull-down assays. *E*, a schematic representation of the Gαi1(91)-RLuc2/mVenus-Gβγ BRET experiment. In the Gαiβγ heterotrimer, the proximity of RLuc2 (fused to Gαi1) to mVenus (fused to Gβγ) generates higher energy transfer (BRET); reduced BRET indicates the dissociation of Gαi1(91)-RLuc2 from mVenus-Gβγ. *F*, change in basal Gαi1(91)-RLuc2/mVenus-Gβγ BRET in HEK293T cells transfected with the indicated GIV-WT or GEM-deficient F1685A (‘FA’) mutants in the same experiment. The average BRET was calculated over 3 min after adding the Rluc2 substrate, Coelenterazine-h, and the corresponding value from GIV-FA (inactive) cells was subtracted. The experiment was performed in three independent biological replicates on different days, each containing three technical replicates. *Error bars* represent SEM (n = 3 biological replicates). The graphs were plotted using GraphPad Prism 5 and statistical significance was calculated using Mann–Whitney paired *t*-test.

Prior characterization of GIV in invertebrate species such as *C. elegans* and *drosophila* described the presence of a PBM on the protein's C-terminal end (18). When we performed a BLAST alignment of the PBM (H_2_N-EYGCV-COOH) found in *C. elegans* and *drosophila* against the vertebrate database, we found that the PBM sequence aligned to several predicted GIV transcripts (Fig. 1*A*, Fig. S1, *A* and *B*). This intrigued us for two reasons: First, this PBM sequence is highly conserved across all vertebrate and invertebrate species analyzed (Fig. S1*B*), suggesting a conserved evolutionary function. Second, despite such conservation, all prior studies on mammalian GIV used constructs lacking this module. It is also worth noting that GIV's PBM sequence also resembles that of Daple's (Fig. 3*A*), a gene that belongs to the same family as GIV, *i.e.*, CCDC88 family (9, 18). This is particularly interesting because Daple, to the best of our knowledge, has not been found in the genome of invertebrates (Figs. 1*A* and S1*A*).

To further characterize GIV's PBM, we first analyzed the expression pattern of GIV (and the other ccdc88 family members) in zebrafish embryos. We chose zebrafish because: (1) it is a vertebrate animal in which all three members of the ccdc88 family exists—A–C; (2) its small size and rapid development allows for analysis of ccdc88 expression in the entire intact animal and across multiple timepoints, and (3) a systematic study on GIV, or any of the ccdc88 family members, in zebrafish has not been done. We noted that GIV is duplicated in zebrafish and is annotated as ccdc88Aa and ccdc88Ab (Fig. 1*A*); such duplication is a frequent event in teleost evolution (20). Whole-mount RNA *in situ* hybridization on zebrafish embryos reveals a ubiquitous expression pattern for all ccdc88 family members at 24 h post fertilization (hpf) (Fig. 1*B*). As development progresses, expression is localized toward the anterior region of the embryo and on structures that appear to be hatching gland cells over the yolk sac at 48 hpf. Only ccc88Ab shows expression onto structures that resemble lateral line hair cells at 96 and 120 hpf (Figs. 1*B* and S1*C*). We selected ccdc88Ab (herein referred to as zGIV) for further studies due to its unique expression pattern and because it contains both the previously defined G-protein regulatory GEM motif and the newly described PBM sequence.

In order to confirm if the conserved GEM and PBM sequence on zGIV can indeed bind to the α-subunit of trimeric Gi-proteins and PDZ proteins, respectively, we overexpressed the C terminus of zGIV (tagged to EGFP) in cells and subjected the cell lysates to interaction assays with purified GST-tagged Gαi3 (Fig. 1*C*) or GST-tagged PDZ domains of ParD3 and Dvl (Fig. 1*D*). The cell polarity regulator, ParD3, and the Wnt signaling regulator, Dvl, were chosen as the PDZ proteins for this study because they have been identified as interactors of GIV in prior studies without a clear understanding of the mode of these interactions (14, 21, 22). In addition, these two PDZ proteins have been demonstrated to bind to Daple's PBM (9, 10, 12, 16). Finally, because of the high sequence similarity between zGIV's and Daple's PBM, we suspected that zGIV's PBM may also bind to ParD3 and Dvl and hence, tested the C terminus of zebrafish Daple (zDaple) alongside zGIV in the same assays. Both proteins bound Gαi3 and PDZ proteins (Fig. 1, *C* and *D*). Furthermore, consistent with what is expected for GEMs, both zGIV and zDaple bound G-proteins in a nucleotide-dependent manner, in that both proteins preferentially bound the inactive conformation of G-proteins (as mimicked by loading the G-protein with GDP), but not the active G-protein conformation (as mimicked by loading the G-protein with both GDP and aluminum fluoride, AlF_4_^−^) (Fig. 1*C*). Surprisingly, we observed that G-proteins bound consistently and significantly less to zGIV compared with zDaple. Overall, these findings indicate that the GEM and PBM sequences on zGIV are functional.

Taken together, we conclude that while GIV's PBM module is functionally conserved in both vertebrates and invertebrates, the absence of a GEM motif in invertebrates suggests sequential evolution of the two functional modules: the ability to bind PDZ proteins *via* the PBM evolved earlier, whereas G-protein binding and activation may have evolved later only among vertebrates. We noted, however, that the GEM sequence in zGIV binds weakly, albeit specifically (in a nucleotide-dependent manner), to Gαi.

### Human GIV has a long transcript isoform (GIV-L) coding for a C-terminal PBM

**Figure 2:**
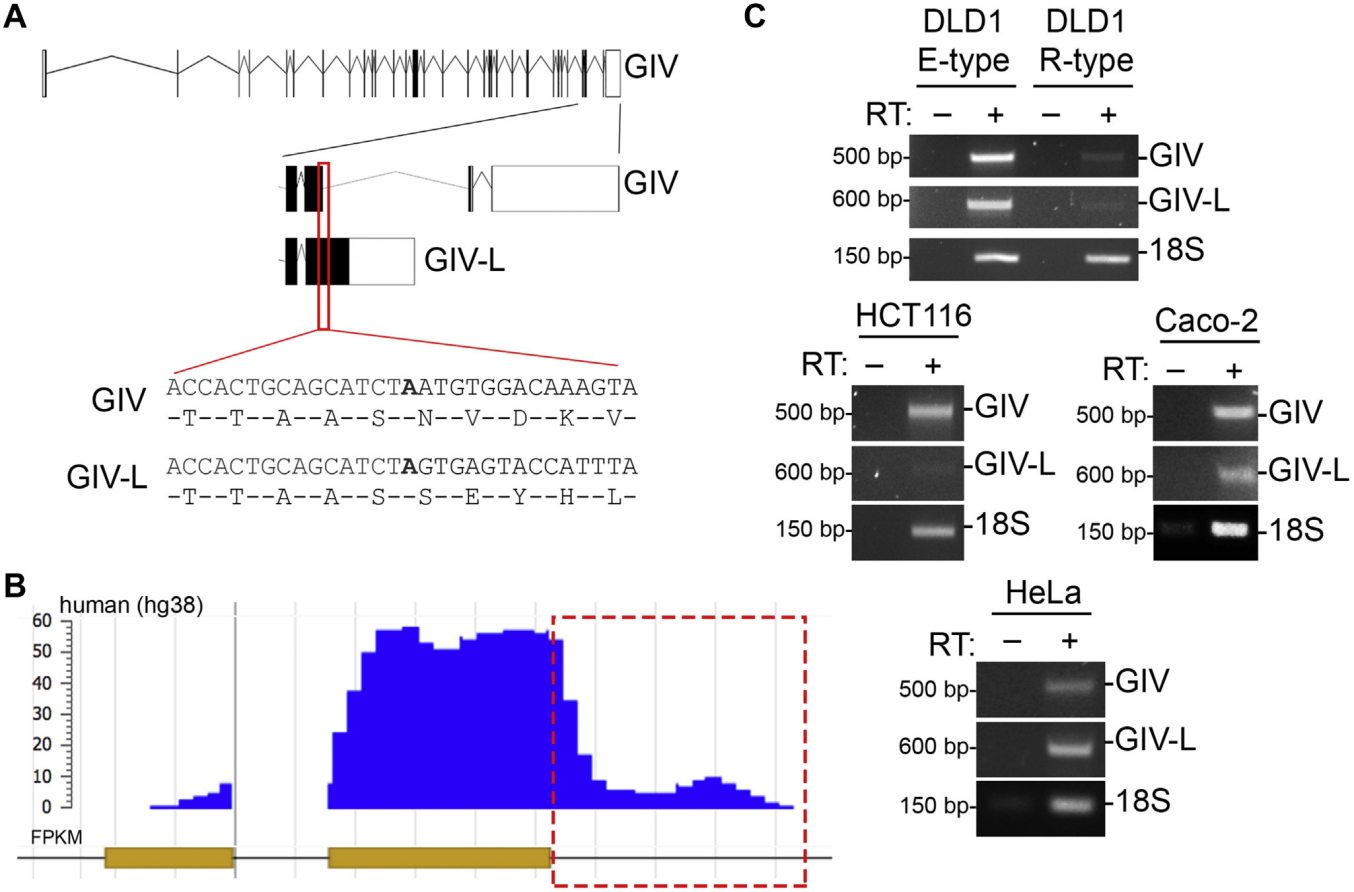
**GIV-L, a human transcript for GIV, translates a variant protein that contains a PBM.***A*, schematic of GIV and GIV-L transcript as annotated in ensembl. Indicated in *red* is region where transcript sequence of GIV (Exon 31) and GIV-L diverges. The *panel below* shows nucleotide sequence and translated sequence of indicated region. *B*, MeRIP-Seq analysis of GIV transcript as annotated in MeT-DB (http://compgenomics.utsa.edu/methylation/) to map degree of m6A-methylated RNA. Highlighted in *red* is the corresponding region of GIV-L transcript (the intron immediately downstream of exon 31) indicating the methylated region. *C*, reverse-transcription PCR of GIV and GIV-L transcript in multiple cell lines: DLD1 E-type and R-type (*top*), HCT116 (*middle*, *left*), Caco-2 (*middle*, *right*), and HeLa (*bottom*).

Several transcriptional isoforms of human GIV are predicted to have a PBM in their translated products (Fig. 1*A* and Fig. S1, *A* and *B*) (XM_005264418.5, XM_017004476.2, XM_017004477.2). A sequence analysis of GIV's transcript revealed that the intron immediately downstream of exon 31 codes for a product containing the PBM (Fig. 2*A*). On the 3′ end of exon 31, a splice event occurs, which processes the nascent transcript into GIV as currently described in the literature (NM_001135597.2) (6, 7). The new isoform of GIV lacks the splice event and, thus, leads to a processed transcript that translates into a larger GIV (by approximately 150 amino acids) protein that contains a PBM, inspiring the nomenclature, GIV-L.

Next, we probed what might be triggers for alternative splicing of GIV into two isoforms. RNA methylation is a frequent event in eukaryotic cells and can affect RNA stability and splicing (23, 24). Furthermore, N^6^-Methyladenosine (m^6^A) modification tends to occur on the last exon of a gene (25). Interestingly, when we analyzed the methylation of GIV in a methyltranscriptome database (MeT-DB V2.0) (26, 27) we observed that the same intronic region downstream of exon 31 is subjected to m^6^A modification (Fig. 2*B*). This analysis supports the notion that mammalian cells have a protein coding transcript for GIV-L.

To validate the prediction, we designed unique primers to GIV and GIV-L (see Experimental procedures for sequence). Among the cell lines tested, we observed that DLD1 E-type, Caco-2, and HeLa cells contain appreciable levels of GIV-L, whereas DLD1 R-type and HCT116 cells do not (Fig. 2*C*). It is noteworthy that among the cancer cell lines tested, DLD1 E-type and Caco-2 cells, and to some extent HeLa cells, form cell–cell junctions, whereas DLD1 R-type and HCT116 do not (28, 29). This suggests the possibility that GIV-L may be expressed in cells with junctions.

### Both GIV and GIV-L can bind Gαi but differ in their abilities to dissociate Gαi/GβƔ trimers in cells

Both GIV and GIV-L contain an identical GEM motif; however, only GIV-L contains a C-terminal PBM (Fig. 3*A*). Therefore, we asked if the presence of the PBM module impacts G-protein binding and if so, how is it affected. We performed a series of biochemical assays that have been used to rigorously validate GIV's ability to bind and activate G-proteins (30). Coimmunoprecipitation experiments confirmed that while both GIV and GIV-L bind G-protein, Gαi3 (Fig. 3*B*), binding appears to be weaker in the case of GIV-L, which is consistent with our observations in pull-down assays (Fig. 1*C*). Furthermore, GST pull-down assays using GST-tagged Gαi3 and cell lysates as sources of GIV or GIV-L further confirmed that while both GIV isoforms bind Gαi3 and require a functional GEM motif to do so (as determined by the loss of binding with a well-characterized GEM-deficient F1685A mutant), G-protein binding in the case of GIV-L appears to be weaker than GIV (Fig. 3, *C* and *C*′). Finally, we sought to determine if GIV-L, like GIV, binds to Gαi3 in a GDP-dependent manner. When GST-tagged Gαi3 was purified and loaded with GDP or GDP-AlF_4_^−^, we observed that GIV-L specifically binds to Gαi3 in the GDP-bound state (Fig. 3, *D* and *D*′). These biochemical studies demonstrate the conserved ability of GIV-L, similar to GIV, to bind to G-proteins *in vitro*.

Next, we studied if GIV-L binds Gαi in cells and how such binding may impact Gαi/GβƔ trimers. Solved structures of GIV-bound Gαi have confirmed that GIV engages the SwII region of Gαi and shares binding determinants with GβƔ (31, 32). These studies provided a structural basis for how GIV displaces GβƔ from Gαi in cells (33). To determine if the binding of GIV-L to Gαi observed *in vitro* translates into G-protein signaling in cells, we utilized a bioluminescence resonance energy transfer (BRET) assay measuring the association between luciferase-tagged Gαi and mVenus-tagged GβƔ (Fig. 3*E*) (34, 35). In this assay, a loss of BRET signal indicates GβƔ dissociation from the G-alpha subunit and, therefore, activation of the G-protein. In HEK293T cells ectopically expressing GIV (wt or F1685A) or GIV-L (wt or F1685A), we observed an expected decrease in the BRET ratio in the presence of GIV (wt) compared with GIV (FA). Surprisingly, GIV-L wt did not lead to a similar decrease in the BRET ratio when compared with its corresponding mutant, GIV-L-FA (Fig. 3*F*). These findings suggested that despite the fact that both GIV and GIV-L have a functional GEM motif, which can bind Gαi *in vitro*, only GIV, but not GIV-L, may trigger G-protein dissociation in cells, at least under the conditions tested.

Although we consistently observe what appears to be a weaker binding between GIV-L and G-proteins, it would be ideal to utilize fragments of purified GIV-L containing the GEM and PDZ module in order to confirm the lower affinity to G-proteins. However, attempts to purify recombinant GIV-L (and zGIV) have been unsuccessful. In *E. coli*, the protein was trapped in inclusion bodies, and despite numerous attempts at solubilization and refolding of the protein from such inclusions, protein precipitation prevented us from getting sufficient high-quality functional protein. To overcome this technical limitation, we proceeded with a multimodality approach instead that involved experimental designs with the full-length endogenous proteins in cells.

### Multiple PDZ proteins bind the PBM on GIV-L and modulate Gα-protein binding

A notable feature of the C-terminal PBM on GIV-L is its high sequence conservation to Daple (Fig. 3*A*). Because prior studies in Daple have shown that its G-protein modulatory function may be allosterically modulated allosterically by binding of PDZ proteins to Daple's PBM (12), we tested if the coexpression of Dvl altered the ability of GIV-L to dissociate Gαi/GβƔ trimers in cells. We found that the BRET ratio was significantly lowered in the presence of Dvl (Fig. 3*F*).

**Figure 4:**
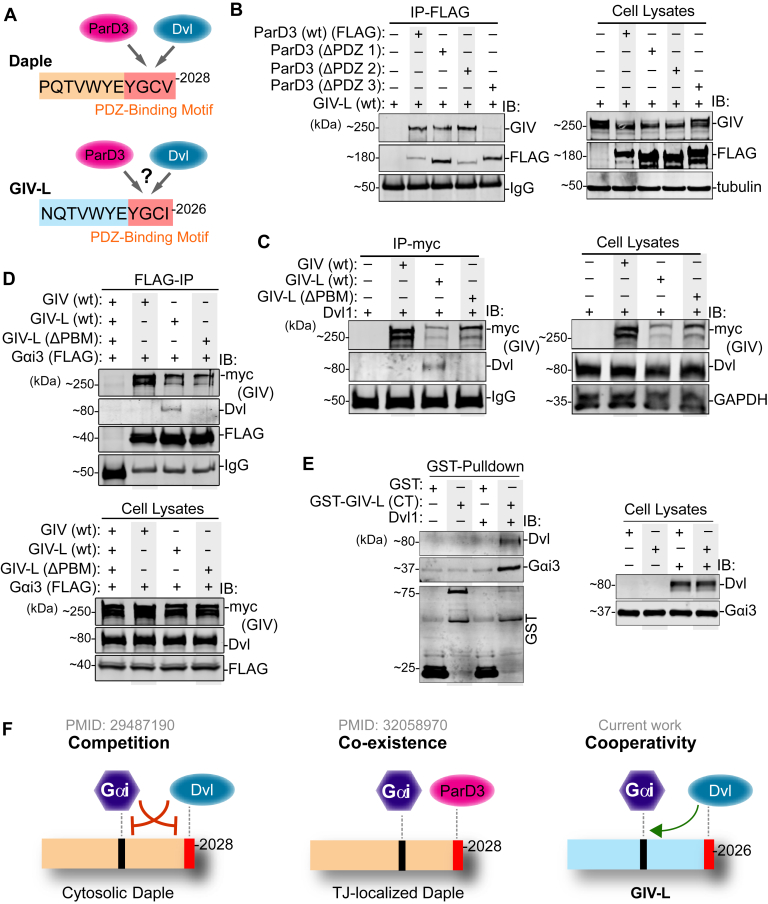
**The PBM motif in GIV-L binds to multiple PDZ proteins and enhances G-protein binding.***A*, schematic depicts the similarities between the sequences of the C-terminal PBMs (highlighted in *red*) of Daple and GIV-L and their respective immediate N-terminal flanking regions. While Daple's PBM is known to bind ParD3 and Dvl, whether GIV-L can bind is tested here. *B*, equal aliquots of lysates of HEK293T cells coexpressing various FLAG-tagged ParD3 constructs and GIV-L (wt) were subjected to immunoprecipitation assays using an anti-FLAG antibody. Bound proteins (*left*) and cell lysates (*right*) were assessed for ParD3 (FLAG) and GIV (myc) by immunoblotting (IB). *C*, equal aliquots of lysates of HEK293T cells coexpressing untagged Dvl1 and either myc-tagged GIV (wt) or GIV-L (wt or ΔPBM) were subjected to immunoprecipitation assays using an anti-myc antibody. Bound proteins (*left*) and cell lysates (*right*) were assessed for Dvl and GIV (myc) by immunoblotting (IB). *D*, equal aliquots of lysates of HEK293T cells coexpressing FLAG-tagged Gαi3 and either myc-tagged GIV (wt) or GIV-L (wt or ΔPBM) were subjected to immunoprecipitation assays using an anti-FLAG antibody. Bound proteins (*top*) and cell lysates (*bottom*) were assessed for Gαi3 (FLAG), GIV (myc), and endogenous Dvl by immunoblotting (IB). *E*, equal aliquots of lysates of HEK293T cells coexpressing untagged Dvl and either GST or GST-tagged GIV-L (CT) was incubated with glutathione agarose beads. Bound proteins (*left*) and cell lysates (*right*) were assessed for Gαi3, Dvl, or GST by immunoblotting (IB). *F*, schematic summarizing the differential impacts of binding of PDZ proteins to the C-terminal PBM motifs in Daple (*left*, *middle*) and GIV-L (*right*) on their ability to bind Gαi protein.

We next dissected the ability of the PBM in GIV-L to bind PDZ proteins and if such binding impacts GIV-L's ability to bind G-proteins. Because Daple's PBM has been shown to bind the PDZ domains of ParD3 and Dvl (Fig. 4*A*) (10, 12), we prioritized these two PDZ proteins in interaction assays with GIV-L Mirroring exactly what was shown for Daple (12), we observed in coimmunoprecipitation studies a specific interaction between GIV-L and the third PDZ domain on ParD3 (Fig. 4*B*). This ability to bind multiple PDZs, and yet preferentially doing so to one but not the other PDZ module on the same protein, highlights a key property of many PBMs. As with Daple, Dvl co-immunoprecipitated exclusively with GIV-L, but not with GIV or a deletion mutant of GIV-L lacking the C-terminal PBM (GIV-L ΔPBM) (Fig. 4*C*). These findings show the similarities between GIV-L's and Daple's PBM and demonstrate that this module is necessary for the GIV-L·PDZ interactions.

Prior work showed that binding of some PDZ proteins to Daple's PBM modulates the ability of Daple to bind G-proteins (Fig. 4*F*). While Dvl competes with Gαi3 for binding Daple, ParD3 does not. Instead, ParD3 is capable of coexisting in a ternary ParD3·Daple·Gαi complex (12, 36). In the case of GIV-L, co-immunoprecipitation assays from cell lysates overexpressing Gαi3 and either GIV or GIV-L (wt or ΔPBM) showed that Dvl can also precipitate with the Gαi3∙GIV complex (Fig. 4*D*). Furthermore, Dvl was not observed to immunoprecipitate with either GIV or the PBM-deficient mutant GIV-L (ΔPBM), indicating that Gαi3 interacts with Dvl only in the presence of GIV-L with an intact PBM. Interestingly, we also observed enhanced binding of GIV-L to Gαi3 in the presence of Dvl (Fig. 4*E*). Because such augmentation was not observed with the F1685A GIV-L mutant (a mutant which cannot bind G-protein; Fig. S2*A*), our findings highlight the modular nature of this protein and cooperativity between Dvl, GIV-L, and Gαi3 (Fig. 4*F*).

Taken together, these findings paint a picture in which PDZ proteins, by virtue of their ability to bind PBMs on GIV-L and Daple fine-tune the G-protein regulatory function of the latter (Fig. 4*F*). Because the PBMs in both proteins are located ∼400 aa downstream of their respective GEM modules, in a stretch that has been deemed to be intrinsically disordered without any semblance to any folded module (8), it is likely that intermodular phenomenon of competition or cooperativity is mediated *via* allosteric mechanisms such as binding-induced conformational changes (Fig. 4*F*). This context-dependent exposure of the GEM module in GIV-L may, in part, be responsible for the observed lack of change in BRET between Gai-RLuc and mVenus-Gbg in cells expressing GIV-L alone and decreased BRET in the presence of Dvl (Fig. 3*F*).

### The PBM on GIV-L is required for localization at cell–cell junctions

**Figure 5:**
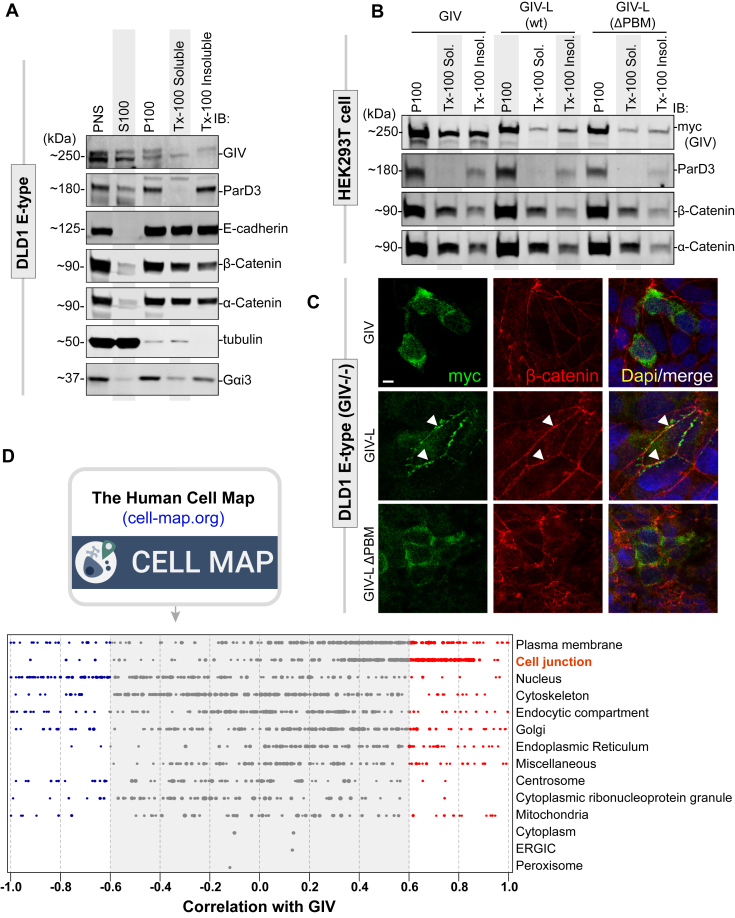
**GIV-L, but not GIV, localizes at cell–cell junctions and its PBM is required for such localization.***A*, DLD1 E-type cells (parental or GIV-knockout) were fractionated into post nuclear supernatant (PNS), cytosolic (S100), crude membrane (P100), membrane detergent soluble (Tx-100 Soluble), and membrane detergent insoluble (Tx-100 Insoluble) pools. Equal proportions of each fraction were assessed for GIV by immunoblotting (IB). Equal loading and reasonable purity (lack of significant cross-contamination) of fractions were confirmed by immunoblotting for ParD3, E-cadherin, β-Catenin, α-Catenin, tubulin, and Gαi3. *B*, cell fractionation studies were carried out as in (*A*) on HEK293T cells exogenously expressing myc-tagged GIV, GIV-L (wt), or mutant GIV-L (ΔPBM). Equal proportion of each fraction was assessed for GIV and other loading and/or fractionation controls (as above) by immunoblotting (IB). *C*, DLD1 E-type cells were transfected with myc-tagged GIV, GIV-L (wt), or mutant GIV-L (ΔPBM), methanol fixed, and stained with anti-myc (green) or anti-β-Catenin antibody. *Arrowheads* indicate cell–cell contact sites. Scale bar, 7.5 μm. *D*, the publicly available BioID-based proximity map of HEK293T cells annotated in the Human Cell Map (HCM) was queried for GIV (without distinguishing GIV and GIV-L) and other preys that cotraffic and localize and functionally associate with GIV (*i.e.*, prey–prey correlations) for various organelle-specific baits. The interactome is enriched for cell-junction-localized proteins.

PDZ proteins are highly enriched in cell junctions where they can serve as docking stations for proteins with PBMs (37, 38, 39). GIV has not only been implicated in regulating cell–cell junction, but it has also been observed to localize to cell junctions *in vivo* (16). While several works have established the importance of Daple's PBM in localizing Daple to cell junctions (11, 12), how GIV localizes there remains elusive. The first clue that GIV-L may be a junction-localized protein comes from cell fractionation studies on DLD1 E-type cell lysates (Fig. 5*A*). Immunoblotting for GIV in these cells revealed the presence of two bands in the post nuclear supernatant (PNS). Both S100 (cytosol) and P100 (membrane) pools derived from the PNS fraction also contain the observed two bands; however, when the P100 fraction was further separated into detergent-soluble and detergent-insoluble fractions, the lower of the two bands was specific to the detergent-soluble fraction while the higher of the two bands was specific to the detergent-insoluble fraction. The detergent-insoluble fraction is known to be enriched in many cell junction proteins because they are typically highly resistant to detergent solubilization (40, 41, 42). The presence of the higher GIV species in the detergent-insoluble pool suggests that this species may be GIV-L.

Next, we ectopically expressed GIV or GIV-L (wt or ΔPBM) into HEK293T cells and performed similar fractionation studies (Fig. 5*B*). When crude membrane (P100) extract was separated into detergent-soluble and detergent-insoluble fractions, we see that GIV equally distributed between the two pools, while GIV-L (wt) has a greater enrichment into the detergent-insoluble pool (Fig. 5*B*). Enrichment was lost when the PBM was truncated. Complementing these fractionation studies, when GIV or GIV-L (wt or ΔPBM) was overexpressed onto DLD1 E-type cells (with endogenous GIV depleted using CRISPR/Cas9) we see that only GIV-L (wt) possessed the ability to localize onto cell–cell junctions, whereas GIV and the PBM-deficient mutant GIV-L (ΔPBM) remained cytosolic (Fig. 5*C*).

Our findings were consistent also with our analysis of the Human Cell Map (HCM) database; HCM is an extensive BioID-based proximity map of HEK293 cells using 192 compartment-specific baits (43). Along with the proximity of prey to these baits, prey cotrafficking, colocalization, and functional associations can be deduced from similarities in prey labeling by the array of baits, *i.e.*, from prey–prey correlations (43). In-depth analysis of the HCM data indicated that GIV is highly correlated with proteins on cell junctions (Fig. 5*D*) and, to a lesser degree, on plasma membranes. Taken together, these observations suggest that GIV-L localizes to cell–cell junctions and that such localization is enabled by its C-terminal PBM, similar to what has been found with Daple.

### BioID-proximity labeling identifies the PDZ-interactome (“PDZome”) of GIV-L

**Figure 6:**
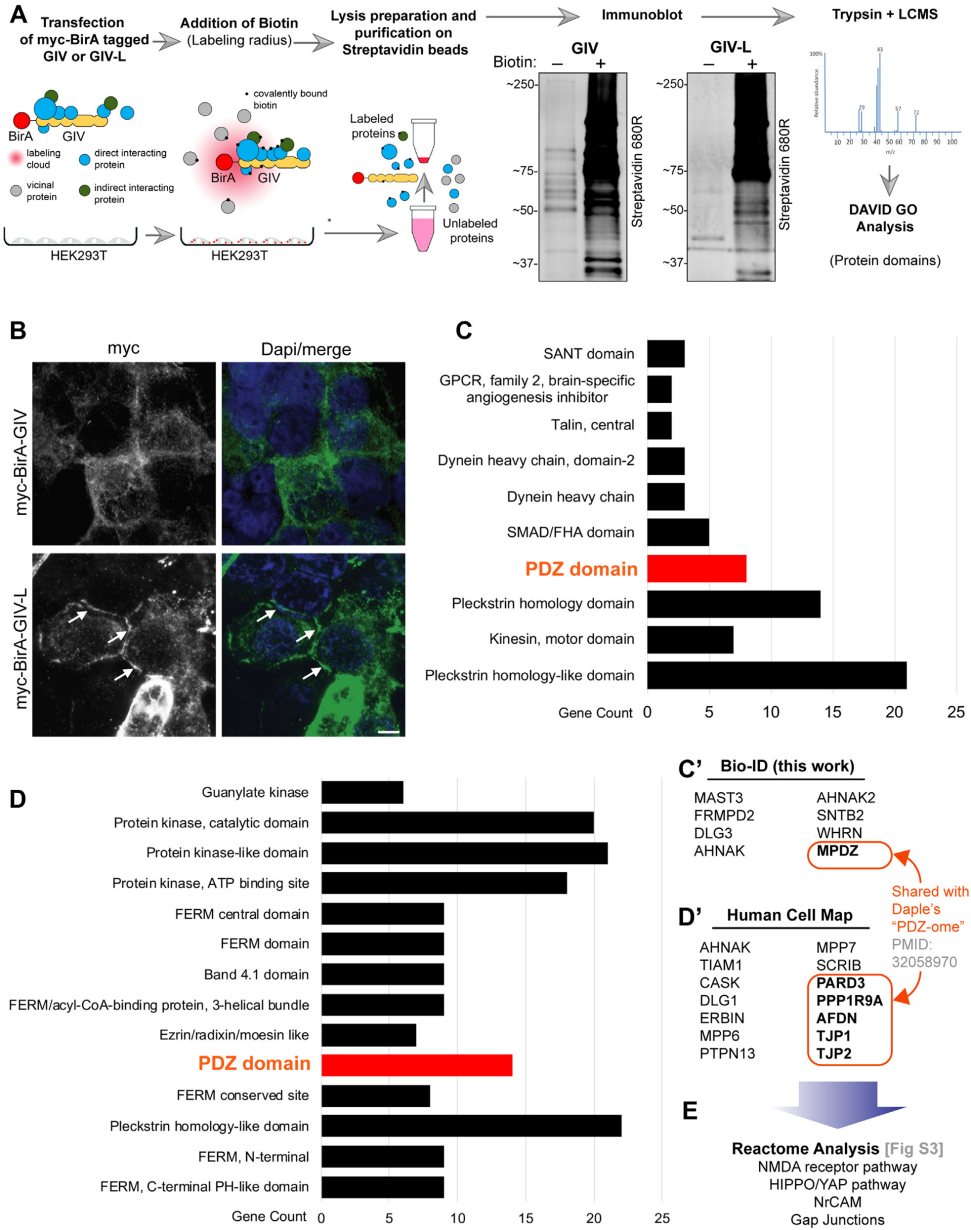
**A protein–protein interaction (BioID) screen identifies the PDZ-interactome of GIV-L.***A*, schematic depicting the key steps in biotin proximity labeling (BioID) studies used to identify the GIV and GIV-L interactomes in HEK293T cells. HEK293T cells were transiently transfected with myc-BirA tagged GIV or GIV-L construct and then treated with free biotin. Equal aliquots of cell lysates were incubated with streptavidin magnetic beads and proteins were eluted by boiling in the presence of excess free biotin. Eluted proteins were analyzed by SDS-PAGE and blotted with AlexaFluor-680-conjugated streptavidin to confirm successful proximity labeling. *B*, HEK293T cells exogenously expressing myc-BirA-tagged GIV or GIV-L were fixed with methanol prior to staining using anti-myc antibody. *Arrows* indicate localization onto points of cell–cell contact. Scale bar, 5 μm. *C*, bar graph summarizing the GIV-L-interacting proteins identified by mass spectrometry and grouped by protein domain using DAVID GO analysis. Top domain categories are shown. *C′*, list of PDZ domain proteins identified. *D*, bar graph summarizing GIV's interactome as annotated in the Human Cell map database and also grouped by protein domain using DAVID GO analysis. Top domain categories are shown. *Panel D′* lists the PDZ-domain containing proteins reported in the Human Cell Map database.

Because the modular composition of large scaffold proteins dictates the protein's interactome, which in turn regulates its localization and functions, we next asked how the GIV interactome changed due to the additional PBM module in GIV-L. To this end, we carried out BioID proximity labeling coupled with mass spectrometry (MS) to identify GIV/GIV-L-interacting proteins (Fig. 6*A*). We validated our BirA-tagged GIV constructs using two approaches: first, we confirmed that these constructs can successfully biotinylate proteins in cells by incubating lysates of transfected cells with streptavidin beads followed by blotting using fluorescent conjugated streptavidin (Fig. 6*A*) and, second, we confirmed that the tagged constructs show the expected subcellular localizations (Fig. 6*B*). In agreement with what was observed in DLD1 E-type cells, GIV-L was found both in cytosol and at cell–cell contact sites, whereas GIV was largely cytosolic in localization.

MS identification and gene ontology analysis (*via* protein domain) of the biotinylated proteins in HEK293T revealed PDZ proteins were proximity labeled by GIV-L, but not by GIV (Fig. 6, *C* and *C*′). Analysis of HCM data using the same gene ontology analysis revealed that there is indeed a set of “PDZome” that interacts with GIV-L (Fig. 6, *D* and *D*′). Furthermore, reactome pathway analysis performed on GIV-L's PDZome identified overrepresentation of NMDA and HIPPO pathways—two pathways that are closely associated with junctional sensing (44, 45, 46).

### GIV is required for contact-dependent growth inhibition, cell–cycle arrest, and apoptosis

While most of the work to date support a pro-oncogenic and prometastatic role for GIV (19, 30, 47, 48, 49), a few have revealed a tumor suppressive function for GIV (50, 51). The pro-oncogenic roles have been demonstrated in stromal cells (Cos7, Vero) or epithelial cells with no (*e.g.*, MDA MB231) or weak junctions (HeLa), whereas tumor suppressive effects were shown in cells with junctions (MDCK, Caco-2, etc.) (13, 50).

**Figure 7:**
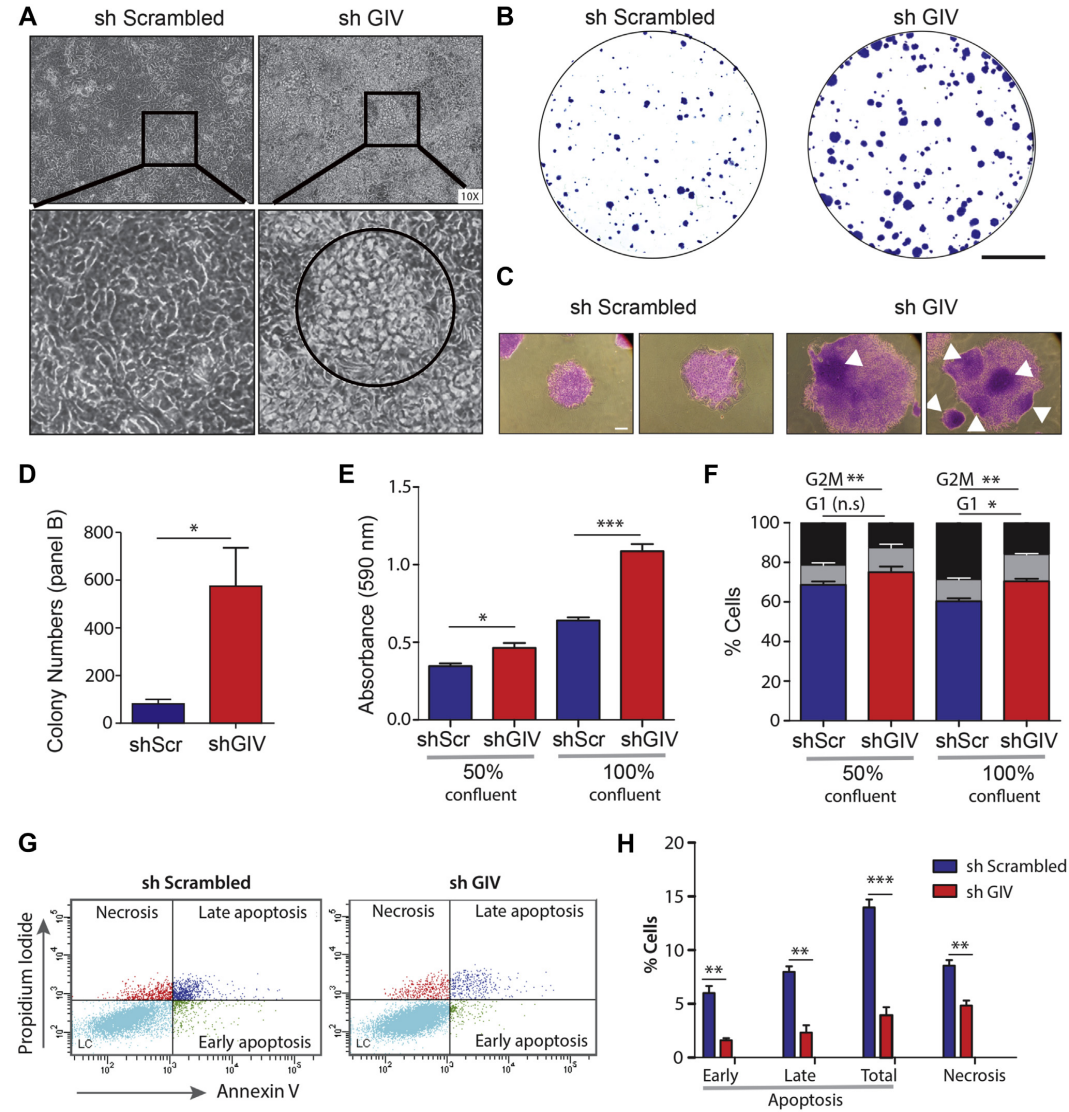
**Depletion of GIV in Caco-2 cells increases anchorage-dependent colony growth, survival, loss of contact-dependent cell-cycle inhibition, and reduced cell death.***A*, phase contrast microscopy images of Caco-2 cells stably expressing a shScrambled or shGIV construct. Caco-2 cells were cultured and grown in a confluent monolayer state for 10 days. *Zoomed-in images* of indicated region are shown below. Central “piling up” of cells is frequently observed in the shGIV monolayer (as outlined). *B–D*, representative images of crystal violet stained colonies, as seen during anchorage-dependent colony growth assays on control (shScrambled) and GIV-depleted (shGIV) Caco-2 cells after 14 days in culture. Scale bar = 10 mm in (*B*). Light microscopy images of representative colonies in (*C*) show the dense areas of piled up cells in shGIV Caco-2 colonies (*arrowheads*). Scale bar = 0.1 mm. Bar graphs (*D*) show quantification of colonies. *Error bars* represent SEM; n = 3 (∗) indicates *p* ≤0.05, as determined by Student's *t*-test. *E*, MTT proliferation assay on control (shScrambled) and GIV-depleted (shGIV) Caco-2 cells grown at 50% or 100% confluency. Bar graphs show quantification of absorbance at 590 nm. *Error bars* represent SEM; n = 3. (∗) indicates *p* < 0.05, and (∗∗∗) indicates *p* < 0.001, as determined by Student's *t*-test. *F*, cell cycle distribution of control (shScrambled) and GIV-depleted (shGIV) Caco-2 cells grown at 50% or 100% confluency. Bar graphs show % of cells in each phase of the cell cycle. *Error bars* represent SEM; n = 3. (∗) indicates *p* < 0.05, (∗∗) indicates *p* < 0.01, n.s., nonsignificant, as determined by Student's *t*-test. *G* and *H*, representative cytograms (*G*) of apoptotic and necrotic control (sh Scrambled) and GIV-depleted (shGIV) Caco-2. The *lower-right* (annexin V^+^PI^−^ cells) and the *upper-right* (annexin V^+^PI^+^ cells) quadrants show early and late apoptotic cells, respectively, while the *lower-left* (annexin V^−^PI^−^ cells) and the *upper-left* (annexin V^−^PI^−^ cells) quadrants represent viable and necrotic cells, respectively. *H*, bar graphs display the % of apoptotic and necrotic cells in (*G*). *Error bars* represent SEM; n = 3. ∗∗*p* < 0.01, ∗∗∗*p* < 0.001, as determined by Student's *t*-test.

The well-differentiated CRC cell line Caco-2 is known to form well-defined junctions, was recently shown to have morphogenesis defects upon GIV depletion (50) and confirmed to express GIV-L (Fig. 2*C*). Therefore, we proceeded with the use of the same GIV-depleted (by shRNA) Caco-2 cell line as a model system for a series of phenotypic studies (50). First, we found that loss of GIV was associated also with a loss of contact-dependent growth arrest, as determined by growth of cells in patches of “piled-up” cells in monolayers (circle; Fig. 7*A*). Second, the same cells produced colonies in larger number and size in anchorage-dependent colony growth assays (Fig. 7, *B*–*D*). When the stained colonies were observed under light microscopy, colonies from GIV-depleted cells were denser and more composed of “piled up” cells (arrowheads; Fig. 7*C*), which is in keeping with our observations in monolayers (Fig. 7*A*). Third, higher colony growth was associated with a higher metabolic activity, as determined by the enhanced ability of GIV-depleted cells to metabolize the tetrazolium dye, MTT, irrespective of confluency (Fig. 7*E*).

To determine if the observed higher growth in GIV-depleted cells was due to merely higher proliferation, or lower cell death, or both, we assessed for the distribution of cells across different stages of the cell cycle (Fig. 7*F*) and for the population of cells undergoing cell death (Fig. 7, *G* and *H*). We observed an increase in G0/G1 phase in GIV-depleted cells at 100% confluency. This increase was accompanied with a concomitant decrease in the distribution of cells in the G2/M phase. When cell death was analyzed under the same conditions, we observed an overall decrease in cell death in GIV-depleted cells (Fig. 7, *G* and *H*). Together, these findings highlight GIV's tumor-suppressive role in the well-differentiated Caco-2 cell line.

### GIV-L, but not GIV, is suppressed during normal to adenoma progression in the colon

**Figure 8:**
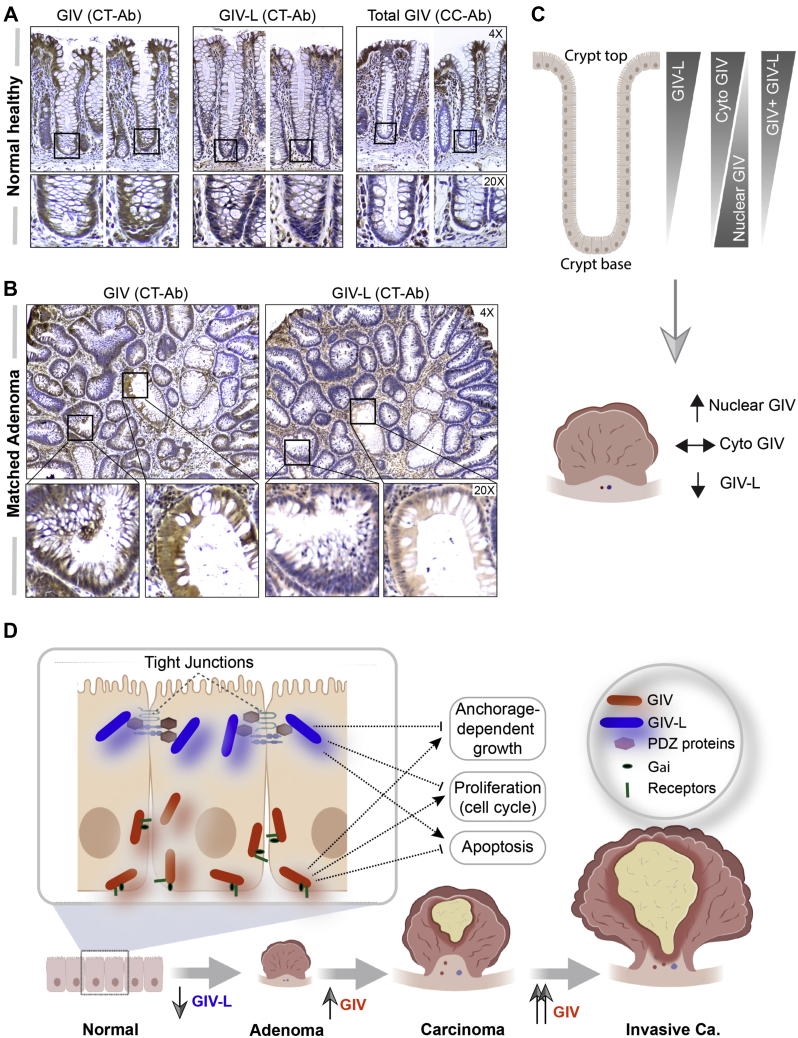
**GIV-L is preferentially expressed in the surface epithelium of colon crypts and is downregulated in the transformed epithelium in colon polyps.***A* and *B*, images representative of patterns of GIV staining, as determined by immunohistochemistry staining on normal healthy human colon (*A*) and matched adjacent adenoma (*B*) with various GIV (total and isoform specific) antibodies. See also Figure S3 for validation studies on the antibodies. *C*, schematic summarizing the observed expression pattern observed in panels *A* (*top*) and *B* (*bottom*). ↑, ↓ and ↔ indicate upregulation, downregulation, and no discernible changes in expression, respectively. *D* and *E*, working model of the opposing roles (*D*) and patterns of altered expression (*E*) of GIV and GIV-L isoforms in the colonic epithelium. Cytosolic GIV promotes stemness, growth, survival, and cell migration, whereas cell-junction-localized GIV-L inhibits growth, survival cell cycle, cell death.

The increase in cell proliferative properties prompted us to examine the expression of GIV and GIV-L in normal and adenoma tissues (Fig. 8, *A* and *B*). To this end, we utilized custom-made antibodies raised against unique epitopes on GIV or GIV-L (Fig. S4*A*). Validation studies confirmed their specificity and ruled out cross-reactivity against isoforms (Fig. S4*B*). Furthermore, they were tested alongside several commercially available antibodies that are expected to detect both isoform (total GIV). In normal healthy colonic tissue (Fig. 8, *A* and *C*), we observed total GIV expression, as determined by GIV (CC-Ab) antibody, ubiquitously throughout the colon epithelial layer. Interestingly, staining with the specific GIV (CT-Ab) and GIV-L (CT-Ab) showed that GIV (CT-Ab) is also ubiquitously expressed (both in cytosolic and nuclear staining); however, GIV-L (CT-Ab) is restricted to the surface epithelial layer. In matched adenoma tissues (Fig. 8, *B* and *C*), we observed an increase in GIV (CT-Ab) levels, but this increase was restricted to nuclei expression. No change in the cytosolic pool was observed. With regard to GIV-L (CT-Ab) staining, we observed a decrease in expression.

These histological observations, taken together with those from our cell-based models, lead us to propose the following working model (see legend; Fig. 8*D*) in which GIV and GIV-L perform opposing functions to maintain epithelial homeostasis and that such functions are dictated based on protein subcellular localization. When GIV is cytosolic, it couples readily with receptors on the basolateral surface and with G-proteins to primarily enhance signals that promote stemness, growth, survival, and cell migration/invasion. By contrast, GIV-L is on cell junction, where it senses junctional integrity and signals to inhibit growth, cell cycle progression and instead, promotes cell death. What those signaling pathways are remains to be determined.

## Discussion

### CCDC88A (GIV/Girdin) shows evolutionary flexibility of modularity

In this work, we confirmed the presence of two isoforms of GIV in vertebrates; besides the isoform that was known to exist and participate in the fine-tuning of endomembrane trimeric GTPase signaling downstream of multiple receptors, we show that there exists another long isoform of GIV, GIV-L, which contains a PBM. The absence or presence of the PBM determines GIV's localization to cell–cell junctions, interactions with PDZ proteins, ability to bind and dissociate Gi trimers using its GEM motif and GIV's overall functions in junction-containing epithelial cells. The presence of the PBM in invertebrates indicates that the motif has been conserved across evolution and not lost, as previously hypothesized (18), suggesting that the PDZ-interacting module on GIV appeared early and remained conserved throughout evolution. By contrast, the GEM motif is absent in invertebrates, but present across all vertebrate species studied, suggesting that the G-protein regulatory module on GIV appeared later in evolution. The zebrafish form of GIV (zGIV) contains both a functional GEM and PBM motif, representing the earliest species in which both modules coexist in the same protein. Furthermore, expression of zGIV is found on lateral line hair cells, a specialized ciliated cell (52, 53). Given the well-established importance of G-proteins and Dvl in cilia function and positioning (54, 55, 56, 57), it is plausible that zGIV regulates G-protein and PDZ interaction in these polarized epithelial cells.

### Modularity dictates localization, interactomes, and function

We also confirmed that the GIV-L transcript also exists in humans and that it contains the PBM and approximately 150 extra amino acids. This longer isoform may have been previously missed for various reasons, including the absence of the transcript in the cDNA library used to identify GIV or due to an incomplete annotation of transcripts at the time the gene was discovered (6, 7). Prior work has shown that GIV localizes to various subcellular compartments, including on cell junctions of mammalian cells (10). Furthermore, GIV was found to interact with PDZ proteins such as Dvl and ParD3 (14, 22), although how such interactions could be mediated remained unsolved. Cell junctions are known to be clustered with PDZ proteins where a complex PDZ-PBM interaction network regulates signaling (37, 38, 39). By showing that GIV-L, and not GIV or a GIV-L mutant that lacks the PBM, localizes to cell junctions and binds to PDZ proteins, we show that both localization and PDZ-binding require GIV-L's PBM. Furthermore, we found that GIV-L transcript was readily detectable in some cell lines, but not others; expression was virtually restricted to epithelial lines that readily make cell–cell junctions. We conclude that between the two isoforms of GIV that coexist in cells, it is GIV-L that fine-tunes junctional signaling through its PBM.

Among the phenotypic readouts investigated here, we focused on key epithelial phenotypes previously studied in the context of GIV. We, and others, have documented on numerous instances that mammalian GIV supports cellular phenotypes, *e.g.*, cell proliferation, growth, survival, migration, and invasion (6, 21, 30, 47, 48, 49). In addition, numerous groups have documented that GIV expression goes up during neoplastic progression in numerous cancers (reviewed in (58)). Based on these observations, GIV has been generally believed to serve primarily as an oncogene that fuels cancer initiation and progression. However, this belief has recently been challenged by others who have suggested a tumor-suppressive role for GIV (50), although the molecular mechanism for such contrasting roles for the same protein was not revealed. By showing here that GIV-L maintains epithelial homeostatic properties (*e.g.*, junction-dependent cell cycle and growth inhibition and apoptosis), we provide the first insights into how GIV may perform opposing roles in epithelial cells *via* its two isoforms. Because cell junctions, in addition to its adhesive properties, are well-regarded as a cellular structure that blocks tumor growth (39), GIV maintains epithelial integrity (13, 14, 50), we propose a working model (see Fig. 8*D*) where the two opposing functions of GIV, *i.e.*, tumor suppressor *versus* oncogene are driven by its subcellular localizations; localization at cell junctions enables GIV-L to exert its tumor-suppressive functions, whereas localization in cytosol enables GIV to access various receptors and G-proteins on basolateral membranes to exert its pro-oncogenic signaling functions. This model is further supported by the detection of GIV-L transcript in colorectal cancer cells that form cell junctions and a lack of transcripts in cells that do not form junctions. That the expression of GIV-L, but not GIV, is suppressed in colon tissue during adenoma formation further supports the model that GIV-L is likely to be the tumor-suppressive isoform.

### CCDC88 proteins exemplify evolutionary enrichment of how PBMs may fine-tune G protein/receptor signaling

It is noteworthy that CCDC88A/GIV is not the only member of the CCDC88 family that has a PBM. CCDC88C/Daple also features a C-terminal PBM motif that is similar but not identical in sequence to that in GIV-L, raising the possibility that GIV-L has a unique PDZ interactome that may not be identical to that of Daple. Preliminary analysis of our own BioID proximity labeling studies, and others, reveals that GIV-L indeed binds to several PDZ domain-containing proteins, which only partially overlaps with that of Daple (12). A more thorough and quantitative BioID and MS study is warranted to definitively conclude the similarities and differences in binding. However, interaction assays confirmed that GIV-L and Daple can indeed bind to some common PDZ-proteins (*e.g.*, ParD3 and Dvl). Surprisingly, prior work has described the interaction of GIV to PDZ containing proteins without directly focusing on GIV-L. Because these works use a coil-coiled antibody (which can recognize both GIV and GIV-L), we believe that co-immunoprecipitated GIV-L in those assays may have confounded the findings. Alternatively, because coil-coiled domains can oligomerize, it is feasible that the GIV construct used in those assays may have interacted with GIV-L in cells.

We also provided evidence for how the PBM·PDZ interactions of members of the CCDC88 family may represent a mechanism *via* which G protein signaling *via* GEM motifs in CCDC88 appears to have been subjected to higher orders of regulatory controls during evolution. For example, by showing that GIV-L bound G-protein Gαi preferably in the presence of Dvl, we demonstrated modular cooperativity. These findings add to the prior examples of competition and coexistence between PBM·PDZ and GEM·G-protein interactions in CCDC88C (12, 36). Why some PDZs cooperate and/or coexist in complexes with CCDC88 and Gαi proteins while others compete remains unknown and warrants further investigation. We speculate that due to GIV-L's localization pattern, it may serve as a scaffold that limits G-protein signaling to cell junctions and thereby G-protein-mediated enhancement of cell proliferation. GIV, on the other hand, remains cytosolic, which may allow it to rapidly localize to other membranes (basolateral and endomembrane compartments) where activation of G-protein may enhance cell proliferation. In others, their relative ratio in cells may offer an opportunity to fine-tune the context of G-protein signaling that is triggered by GIV.

In conclusion, we have identified a novel isoform of GIV that contains an evolutionarily conserved PBM and demonstrate how evolutionary flexibility between two isoforms of GIV—one without and one with PBM—dictates protein localization, interactome, and functions. Insights into how binding to PDZ proteins shapes GIV's localization and interactions have revealed how modularity regulates GIV's functions. Such revelation can help us to further understand the role GIV plays in tissue homeostasis and how its dysregulation may trigger diseases.

## Experimental procedures

### Cell lines and culture methods

DLD1 cells were cultured using RMPI media containing 10% FBS. Cells were routinely passaged at a dilution of 1:5 to 1:10. HEK293T, Caco-2, and HeLa cells were cultured using DMEM media containing 10% FBS and routinely passaged at a dilution of 1:5 to 1:10. HCT116 cells were cultured in McCoy's 5a Medium containing 10% FBA and passed at a dilution of 1:5 to 1:10. Caco-2 (Sh Scrambled and Sh GIV) cultured as previously described (50).

### Zebrafish Husbandry

Zebrafish protocol and maintenance were performed using methods approved by the University of California, San Diego Institutional Animal Care and Use Committee (UCSD-IACUC). Zebrafish wild-type (AB) strains were used for tissue expression studies.

### RNA probe synthesis and whole-mount RNA *in situ* hybridization

DNA template used in the *in vitro* transcription of RNA probes was amplified from a pooled cDNA library of zebrafish embryos (12–72 h post fertilization). Flanking the reverse primer was a T7 RNA polymerase site. PCR amplicon was separated on an agarose gel and then extracted using a DNA gel extraction kit (Zymo). Purified DNA was used in an *in vitro* transcription reaction using T7 RNA polymerase (Promega) and DIG RNA labeling mix (Roche). Transcription was carried out at 37 °C for 2 h, followed by the addition of DNase to degrade the template. EDTA was added to ensure a full stop of the transcription reaction. mRNA probe was purified using lithium chloride precipitation and diluted in hybridization buffer (50% formamide, 750 mM NaCl, 75 mM sodium citrate, 50 µg/ml Heparin, 5 mM EDTA, 0.5 mg/ml rRNA, 1 mM Citric Acid, 0.1% Tween-20, pH = 6.0) prior to use in whole-mount RNA *in situ* hybridization.

Whole-mount *in situ* hybridization was performed using the labeled probed as previously described (59). Briefly, embryos were first fixed overnight at 4 °C using 4% paraformaldehyde (PFA) in PBS. After fixation, PFA was washed four times (15 min each) with PBS, followed by two washes (5 min each) with 100% Methanol. At this point, bleaching was performed to remove pigmentation, if necessary. Embryos were rehydrated using successive washes of PBT (0.2% BSA and 0.2% Tween-20 in 1X PBS). Embryos were then treated with proteinase K, washed using PBT, and then equilibrated into hybridization buffer at 65 °C for 2 h. Afterward, embryos were when transferred to hybridization buffer containing DIG-labeled RNA probe and allowed to incubate overnight at 65 °C. Excess probed was removed through excessive washes using hybridization buffer, followed by a gradient of SSC solution (150 mM NaCl, 15 mM sodium citrate) and PBT buffer washes. Incubation with anti-DIG secondary antibody was carried out overnight at 4 °C followed by development using Nitro Blue Tetrazolium and 5-bromo-4-chloro-3-indolyl-phosphate (BCIP). Alkaline-phosphatase reaction was stopped through excessive washes using PBT, and embryos were immediately mounted on methylcellulose and imaged using a light microscope.

### Recombinant protein purification

GST-tagged proteins were expressed in *E. coli* strain BL21 (DE3) and purified. Cultures were induced using 1 mM IPTG overnight at 25 °C. Cells were then pelleted and resuspended in GST lysis buffer (25 mM Tris-HCl, pH 7.5, 20 mM NaCl, 1 mM EDTA, 20% (vol/vol) glycerol, 1% (vol/vol) Triton X-100, 2× protease inhibitor cocktail). Cells were lysed by sonication, and lysates were cleared by centrifugation at 12,000*g* at 4 °C for 30 min. Supernatant was then affinity purified using glutathione-Sepharose 4B beads (GE Healthcare), followed by elution, overnight dialysis in PBS, aliquoted and then stored at –80 °C.

### *In Vitro* GST-Pull-down and *in cellulo* co-immunoprecipitation (CoIP) assays

Purified GST-tagged proteins from *E. coli* were immobilized onto glutathione-Sepharose beads and incubated with binding buffer (50 mM Tris-HCl [pH 7.4], 100 mM NaCl, 0.4% [v:v] Nonidet P-40, 10 mM MgCl_2_, 5 mM EDTA, 2 mM DTT) for 60 min at room temperature. For the pull-down of protein–protein complexes from cell lysates, cells were first lysed in cell lysis buffer (20 mM HEPES, pH 7.2, 5 mM Mg-acetate, 125 mM K-acetate, 0.4% Triton X-100, 1 mM DTT, 500 μM sodium orthovanadate, phosphatase inhibitor cocktail [Sigma-Aldrich], and protease inhibitor cocktail [Roche]) using a 28G needle and syringe, followed by centrifugation at 10,000*g* for 10 min. Cleared supernatant was then used in binding reaction with immobilized GST-proteins for 4 h at 4 °C. After binding, bound complexes were washed four times with 1 ml phosphate wash buffer (4.3 mM Na2HPO4, 1.4 mM KH2PO4, pH 7.4, 137 mM NaCl, 2.7 mM KCl, 0.1% (v:v) Tween 20, 10 mM MgCl_2_, 5 mM EDTA, 2 mM DTT, 0.5 mM sodium orthovanadate). Bound proteins were then eluted through boiling at 100 °C in the sample buffer.

For CoIP assays, cells lysates (as prepared above) were incubated with capture antibodies for 3 h at 4 °C, followed by the addition of Protein A or Protein G beads to capture antibody bound protein–protein complexes. Bound proteins were then eluted through boiling at 100 °C in the sample buffer.

### Gene transcript detection

To generate cDNA for PCR analysis, total RNA was first isolated from cells using TRIzol Reagent (Life Technologies) following manufacturer's protocol. Next, cDNA was reverse transcribed using qScript cDNA SuperMix (QuantaBio). cDNA was then used in PCR reactions with GIV (Fwd: GGAAAACCTACACCAGGCAC Rev: TGCCTGCTCTATTCACGAAGG) or GIV-L (Fwd: TGGAAGTGAAGTTGTTACTC Rev: CACAAGAACCTATAGTATGTG) specific primers. Following PCR, amplicons were analyzed by agarose gel electrophoresis. Due to the large size of the amplicons (over 500 bp) that supersede the optimal limits for qPCR studies, analysis by real-time quantitative PCR was not feasible.

### BRET-based assessment of Gai/GβƔ dissociation

mVenusCT-hGBB1 and mVenusNT-hGBG2 were a gift from Nevin Lambert, Univ. of Alberta (35). pcDNA3.1(+)-hGai1(91)-RLuc2 was generously shared by Michel Bouvier (34).

On day 1, HEK293T cells were plated at a density of 3.5 × 10^5^ cells per well into a 12-well plate using DMEM containing 10%FBS. On day 2, cell culture media was replaced with fresh media and then cells were transfected with 0.2 ⎧g/well CXCR4, 0.2 ⎧g/well VenusCT-Gβ, 0.2 ⎧g/well VenusNT-GƔ, 20 ng/well Gai(91)-Rluc2, and 0.4 ⎧g/well of GIV variants or pcDNA vector control using TransIT-X2 transfection reagent (MirusBio) according to manufacturer's instruction. On day 3, cells were lifted by pipetting and transferred into 1.5 ml microcentrifuge tubes, spun down, and resuspended in DMEM+10% FBS to 40,000 cells/ml. In total, 100 μL of the cell suspension was then replated on a poly-D-lysine-coated 96-well black/clear bottom plate and allowed to adhere. On day 4, cell culture media was carefully removed and replaced with 80 μL of serum-free assay buffer (PBS + 0.1% glucose) for 60 min. The luciferase substrate, coelenterazine-h (10 μM final), was added to each well. The plate was incubated at room temperature for 5 min, after which repeated readings of light emission at 485 and 515 nm were initiated using the Victor X luminescence plate reader (PerkinElmer) over the course of 3 min. The average BRET was calculated over 3 min after adding Coelenterazine-h. The experiment was repeated in three independent biological replicates on different days, each containing three technical replicates. An average of the three biological replicate is shown, and graphs were plotted using GraphPad Prism 5.

### Cell fractionation

Cells were harvested and suspended in homogenization buffer (10 mM sodium phosphate buffer [pH 7.2], 1 mM MgCl_2_, 30 mM NaCl, 1 mM DTT, and 0.5 mM phenylmethylsulfonyl fluoride [PMSF], supplemented with protease and phosphatase inhibitors), and homogenized using a 30-gauge needle and syringe. Unlysed cells were cleared by centrifugation at 1000*g* for 10 min at 4 °C and collecting supernatant. Crude membranes were separated from the homogenate by centrifugation of post-nuclear supernatant at 100,000*g* for 60 min at 4 °C in a TLA-41 fixed-angle rotor in a TLA-100 table-top ultracentrifuge (Beckman Coulter). Pelleted membranes were washed in a homogenization buffer before resuspension in a cell lysis buffer containing 0.4% Tx-100.

### Quantitative immunoblotting

For immunoblotting, protein samples were boiled in a Laemmli sample buffer, separated by SDS-PAGE and transferred onto 0.4μm PVDF membrane (Millipore) prior to blotting. Post transfer, membranes were blocked using 5% Non-fat milk or 5% BSA dissolved in PBS. Primary antibodies were prepared in blocking buffer containing 0.1% Tween-20 and incubated with blots, rocking overnight at 4 °C. After incubation, blots were incubated with secondary antibodies for 1 h at room temperature, washed, and imaged using a dual-color Li-Cor Odyssey imaging system.

### Immunofluorescence and confocal microscopy, image analysis

Cells were fixed using –20 °C methanol (or 4 °C paraformaldehyde, PFA) for 20–30 min, rinsed with PBS, then permeabilized for 1 h using blocking/permeabilization buffer (0.4% Triton X-100 and 2 mg/ml BSA dissolved in PBS). Primary antibody and secondary antibody were diluted in a blocking buffer and incubated with cells for 1 h each. Coverslips were mounted using Prolong Gold (Invitrogen) and imaged using a Leica SPE CTR4000 confocal microscope.

### Image processing

All images were processed on ImageJ software (NIH) and assembled into figure panels using Photoshop and Illustrator (Adobe Creative Cloud). Some images were created using BioRender.com. All graphs were generated using Excel (Microsoft) or GraphPad Prism.

### GIV CRISPR/Cas9 gene editing and validation

GIV guide DNA sequence was cloned into PX-459 vector and transfected into cells using PEI. For selection, puromycin was added to cells, and when untransfected control plates showed 95–100% cell death, cells were washed with PBS and media (without puromycin) was added to cells for 8 h. Following recovery, cells were resuspended and sparsely plated (approximately 30 cells/plate) onto 10 cm plates so that individual cell colonies could be isolated and picked into 12-well plates for screening.

To identify cell clones harboring mutations in gene coding sequence, genomic DNA was extracted using 50 mM NaOH and boiling at 95 °C for 60 min. After extraction, pH was neutralized by the addition of 10% volume 1.0 M Tris-pH 8.0. The crude genomic extract was then used in PCR reactions with primers flanking the targeted site. Amplicons were analyzed for insertions/deletions (indels) using a TBE-PAGE gel. The Indel sequence was determined by cloning amplicons into a TOPO-TA cloning vector (Invitrogen) following the manufacturer's protocol.

### Biotin proximity labeling

BioID was performed as previously described (12). Briefly, HEK293T were plated 24 h prior to transfection with mycBirA-tagged GIV construct. Thirty hours post transfection, cells were treated with 50 ⎧M biotin (dissolved in culture media) for 16 h. Cells were then rinsed two times with PBS and lysed by resuspending in lysis buffer (50 mM Tris, pH 7.4, 500 mM NaCl, 0.4% SDS, 1 mM dithiothreitol, 2% Triton X-100, and 1× Complete protease inhibitor) and sonication in a bath sonicator. Cell lysates were then cleared by centrifugation at 20,000*g* for 20 min, and supernatant was then collected and incubated with streptavidin magnetic beads overnight at 4 °C. After incubation, beads were washed twice with 2% SDS, once with wash buffer 1 (0.1% deoxycholate, 1% Triton X-100, 500 mM NaCl, 1 mM EDTA, and 50 mM HEPES, pH 7.5), followed with once wash using wash buffer 2 (250 mM LiCl, 0.5% NP-40, 0.5% deoxycholate, 1 mM EDTA, and 10 mM Tris, pH 8.0), and once with 50 mM Tris pH 8.0. Biotinylated complexes were then eluted using a sample buffer containing excess biotin and heating at 100 °C. Prior to MS identification, eluted samples were run on SDS-PAGE and proteins were extracted by in gel digest.

### In gel digest

Protein digest and MS were performed as previously described (60). Briefly, the gel slices were cut into 1 mm × 1 mm cubes, destained three times by first washing with 100 μl of 100 mM ammonium bicarbonate for 15 min, followed by the addition of equal volume acetonitrile (ACN) for 15 min. The supernatant was collected, and samples were dried using a speedvac. Samples were then reduced by mixing with 200 μl of 100 mM ammonium bicarbonate-10 mM DTT and incubated at 56 °C for 30 min. The liquid was removed and 200 μl of 100 mM ammonium bicarbonate-55 mM iodoacetamide was added to gel pieces and incubated, covered at room temperature for 20 min. After the removal of the supernatant and one wash with 100 mM ammonium bicarbonate for 15 min, equal volume of ACN was added to dehydrate the gel pieces. The solution was then removed, and samples were dried in a SpeedVac. For digestion, enough solution of ice-cold trypsin (0.01 μg/μl) in 50 mM ammonium bicarbonate was added to cover the gel pieces and set on ice for 30 min. After complete rehydration, the excess trypsin solution was removed, replaced with fresh 50 mM ammonium bicarbonate, and left overnight at 37 °C. The peptides were extracted twice by the addition of 50 μl of 0.2% formic acid and 5% ACN and vortex mixing at room temperature for 30 min. The supernatant was removed and saved. A total of 50 μl of 50% ACN-0.2% formic acid was added to the sample and vortexed again at room temperature for 30 min. The supernatant was removed and combined with the supernatant from the first extraction. The combined extractions are analyzed directly by liquid chromatography (LC) in combination with tandem mass spectroscopy (MS/MS) using electrospray ionization.

### LC-MS analysis

Trypsin-digested peptides were analyzed by ultrahigh-pressure liquid chromatography (UPLC) coupled with tandem mass spectroscopy (LC-MS/MS) using nanospray ionization. The nanospray ionization experiments were performed using a Orbitrap fusion Lumos hybrid mass spectrometer (Thermo) interfaced with nanoscale reversed-phase UPLC (Thermo Dionex UltiMate 3000 RSLC nano System) using a 25 cm, 75-micron ID glass capillary packed with 1.7-μm C18 (130) BEH beads (Waters corporation). Peptides were eluted from the C18 column into the mass spectrometer using a linear gradient (5–80%) of ACN (Acetonitrile) at a flow rate of 375 μl/min for 1 h. The buffers used to create the ACN gradient were: Buffer A (98% H_2_O, 2% ACN, 0.1% formic acid) and Buffer B (100% ACN, 0.1% formic acid). Mass spectrometer parameters are as follows; an MS1 survey scan using the orbitrap detector (mass range (m/z): 400–1500 (using quadrupole isolation), 120,000 resolution setting, spray voltage of 2200 V, ion transfer tube temperature of 275 °C, AGC target of 400,000, and maximum injection time of 50 ms was followed by data dependent scans (top speed for most intense ions, with charge state set to only include +2–5 ions, and 5 s exclusion time, while selecting ions with minimal intensities of 50,000 at which the collision event was carried out in the high-energy collision cell (HCD Collision Energy of 30%), and the fragment masses were analyzed in the ion trap mass analyzer (with ion trap scan rate of turbo, first mass m/z was 100, AGC Target 5000 and maximum injection time of 35 ms). Protein identification and label-free quantification were carried out using Peaks Studio 8.5 (Bioinformatics solutions Inc) Search parameters are as outlined in Table 1 (see supporting information).

### Gene ontology analysis

Proteins identified by MS in biotin-treated samples, but not in non-biotin-treated samples, were analyzed using DAVID. Functional annotation was grouped by INTERPRO protein domains for GO analysis. Classification with *p*-value less than 0.5 was considered as significant.

### Using Human Cell Map for the identification of potential interactors and subcellular compartment annotation of GIV

The HCM data set was downloaded (accessed 01/06/2020), reprocessed, and rescored using SAINTexpress (61) with a modified negative control set only containing untransfected cells. Control cells expressing cytoplasmic BirA∗-FLAG or BirA∗-FLAG-GFP (included in the original analysis (43)) were removed. This was done to eliminate nonspecific proteins that bound to streptavidin beads even in the absence of biotinylation, while retaining possible specific interactions in the cytoplasm. Following rescoring, a 1% Bayesian FDR cutoff was used to filter for confident bait–prey pairs and the resulting data set was used to calculate prey–prey correlations with GIV, as per Go *et al.* (43). Annotation of protein subcellular localization was taken from the Nonnegative Matrix Factorization method used in HCM (43).

### Anchorage-dependent colony growth assay

Anchorage-dependent growth was monitored on regular tissue culture plastics by seeding cells at a density of 5000 cells per well in a six-well plate and incubation for approximately 21 days in 10% FBS media. Media was changed approximately every 3 days to ensure the health of the cells. Cells were then fix and permeabilized using 100% methanol prior to staining with 0.1% crystal violet. Colony growth was imaged by light microscopy and colonies were counted using ImageJ (NIH).

### Cell cycle, apoptosis, and cell proliferation assay

Cell cycle analysis and apoptotic cell quantification were performed using the Guava cell cycle reagent (Millipore Sigma) or the annexin V/propidium iodide (PI) staining kit (Thermo Fisher Scientific), respectively, according to the manufacturer's instructions. Cells were quantified on a BD LSR II flow cytometer and analyzed using FlowJo software (FlowJo).

Cell proliferation was measured using the MTT reagent and cells cultured in 96-well plates. Cells were incubated with MTT for 4 h at 37 °C. After incubation, culture media was removed and 150 μl of DMSO was added in order to solubilize the MTT formazan crystals. Optical density was determined at 590 nm using a TECAN plate reader. At least three independent experiments were performed.

### Immunohistochemistry

Slides containing normal colon and adjacent cancer tissue were deparaffinized in xylene and then rehydrated in a gradation of alcohols to water. Slides were immersed in sodium citrate buffer (pH 6.0) and pressure cooked for 3 min and 30 s for antigen retrieval. Endogenous peroxidase activity was blocked by incubation using hydrogen peroxide. To block nonspecific protein binding, 2.5% goat serum was used. Tissues were then incubated with primary antibodies for 1 h at room temperature and in a humidified chamber. Afterward, slides were rinsed three times with PBS (5 min each rinse). Sections were then incubated with horse anti-rabbit HRP-conjugated secondary antibody for 30 min at room temperature and then washed three times with PBS (5 min each rinse), followed by development with DAB substrate and counterstain with hematoxylin. After development, slides were dehydrated using a gradient of alcohol washes, cleared in xylene, and then mounted with coverslips. Epithelial and stromal components of tumors were identified by staining duplicate slides in parallel with hematoxylin and eosin and visualizing by light microscopy.

### Statistical analysis and replicates

Student's *t*-test was used to determine significance with *p* values of <0.05 set as the minimal threshold for statistical significance. Where statistical analysis was performed, experiments were performed (at least) in triplicates.

## Data availability

The MS proteomics data have been deposited to the ProteomeXchange Consortium *via* the PRIDE partner repository with the data set identifier PXD022601 (62).

## Supporting information

This article contains supporting information.

## Conflict of interest

The authors declare that they have no conflicts of interest with the contents of this article.
