# Supplementary material for: A long isoform of GIV/Girdin contains a PDZ-binding module that regulates localization and G-protein binding
Source: J Biol Chem. 2021 Mar 3;296:100493. doi: 10.1016/j.jbc.2021.100493 (PMC8042451; doi:10.1016/j.jbc.2021.100493)
Supplement: Figures S1 to S4; Table 1 [file mmc1.pdf]

SUPPORTING INFORMATION

A long isoform of GIV/Girdin contains a PDZ binding module that regulates localization and G-protein binding

Jason Ear[1,2 §], Amer Ali Abd El-Hafeez[1, ¶], Suchismita Roy[3], Tony Ngo[3], Navin Rajapakse[1], Julie Choi[1], Soni Khandelwal[4], Majid Ghassemian[5], Luke McCaffrey[6,7], Irina Kufareva[3], Debashis Sahoo[4], Pradipta Ghosh[1, 8-10 §]

[1]Department of Cellular and Molecular Medicine, University of California San Diego, La Jolla, California 92093
[2]Biological Sciences Department, California State Polytechnic University, Pomona, California 91768
[3]Skaggs School of Pharmacy and Pharmaceutical Sciences, University of California San Diego, La Jolla, California 92093
[4]Department of Pediatrics, University of California San Diego, La Jolla, California 92093
[5]Department of Chemistry and Biochemistry, University of California San Diego, La Jolla, California 92093
[6]Rosalind and Morris Goodman Cancer Research Centre, McGill University, Montreal, Canada
[7]Gerald Bronfman Department of Oncology, McGill University, Montreal, Canada.
[8]Department of Medicine, University of California San Diego, La Jolla, California 92093
[9]Rebecca and John Moore Comprehensive Cancer Center, University of California San Diego, La Jolla, California 92093
[10]Veterans Affairs Medical Center, La Jolla, CA.

[§]Corresponding Author. prghosh@ucsd.edu (P.G.), Jear@cpp.edu (J.E.)

SUPPLEMENTARY FIGURE LEGENDS

Figure S1 – Supplement to Figure 1
The C-terminus of GIV has an evolutionarily conserved functional PDZ-binding motif downstream of its G protein binding and/or modulatory domains.

A) Table summarizing the characterized modules and motifs in GIV and Daple. “P” indicates presence, “X” indicates no description, “-” indicates absence.

B) Amino acid alignment of the PBM across various species.

C) A magnified image of a zebrafish stained for zGIV (CCDC88Ab; from Figure 1C) is shown. Scale bar = 1mm

Figure S2- Supplement to Figure 4

The PBM motif in GIV-L is functional and binds PDZ-proteins ParD3 and Dvl.

A-C) GST-pulldown assays were carried out using purified GST-tagged PDZ domains of ParD3 (A) or Dvl (B). Bound proteins were visualized (left) and equal loading of cell lysates (right) were confirmed by immunoblotting (IB). Lysates of HEK293T cells (C) exogenously expressing myc-tagged GIV (wt or F1685A) or GIV-L (wt, F1685A, ∆PBM, or F1685A/∆PBM double mutant) that were used as source of GIV proteins for the pulldown assays.

Figure S3 – Supplement to Figure 6

GIV-L's 'PDZ-ome' provides clues into specific junction-sensing pathways GIV-L may modulate.

Reactome pathway analysis was performed on the GIV-L bound 'PDZ-ome' (**Fig 6D'-E'**) and findings are visualized as ReacFoam (top) or table of statistically enriched pathways (bottom). Boxed regions on top are magnified. Arrows (red) highlight the overrepresentation of two specific junction-sensing pathways, NMDA and HIPPO.

Figure S4 – Supplement to Figure 8

Validation studies for customized rabbit polyclonal antibodies used to detect GIV and GIV-L individually.

A) Schematic depicts the ectopically expressed GIV or GIV-L construct in HEK293T cells and the binging region of the antibodies used.

B) Various GIV antibodies was used to immunoblot HEK293T cell lysates overexpressing EGFP-tagged GIV or GIV-L. EGFP-tagged GIV-L (CT) was overexpressed in HEK293T cells and purified using an anti-GFP camelid antibody. Purified protein, along with cell lysates, was used in SDS-PAGE and western blotting analysis to validate specificity of GIV antibodies.

SUPPLEMENTARY FIGURES

Figure S1

A

	HOOK		Coiled-coil		GBD		GEM		PBM	
**Drosophila**	✓	-	✓	-	X	-	X	-	✓	-
**C. elegans**	✓	-	✓	-	✓	-	X	-	✓	-
**Zebrafish**	✓	✓	✓	✓	✓	✓	✓	✓	✓	✓
**Lizard**	✓	✓	✓	✓	✓	✓	✓	✓	✓	✓
**Chicken**	✓	✓	✓	✓	✓	✓	✓	✓	✓	✓
**Dog**	✓	✓	✓	✓	✓	✓	✓	✓	X	✓
**Mouse**	✓	✓	✓	✓	✓	✓	✓	✓	X	✓
**Monkey**	✓	✓	✓	✓	✓	✓	✓	✓	X	✓
**Human**	✓	✓	✓	✓	✓	✓	✓	✓	X	✓
	GIV	Daple	GIV	Daple	GIV	Daple	GIV	Daple	GIV	Daple

B

```
Homo sapiens                QTVWYEYGCI
Pan troglodytes             QTVWYEYGCI
Rattus norvegicus           QTVWYEYGCI
Mus musculus                QIVWYEYGCI
Gallus gallus               KTVWYEYGCV
Pogona vitticeps            QTIWYEYGCV
Xenopus laevis              QSIWYEYGCV
Danio rerio                 DGLWYEYGCV
Drosophila melanogaster     NSIWYEYGCV
Caenorhabditis elegans      STIWHEYGCV
                            ...*.****.
```

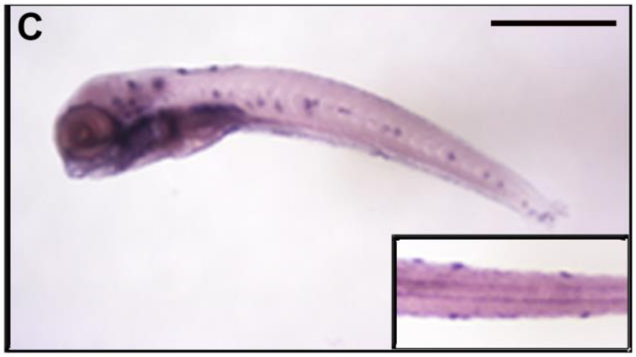

Figure S2

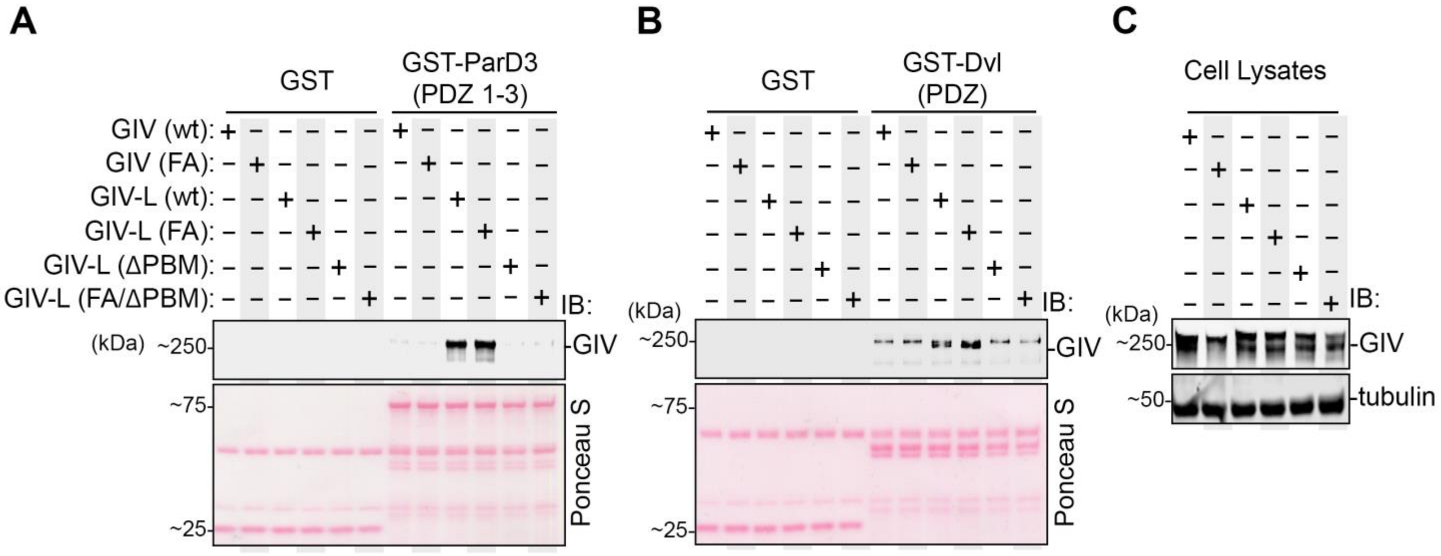
A
GST
GST-ParD3 (PDZ 1-3)
GIV (wt):
GIV (FA):
GIV-L (wt):
GIV-L (FA):
GIV-L (ΔPBM):
GIV-L (FA/ΔPBM):
IB:
(kDa)
~250
GIV
~75
~25
Ponceau S
B
GST
GST-Dvl (PDZ)
IB:
(kDa)
~250
GIV
~75
~25
Ponceau S
C
Cell Lysates
IB:
(kDa)
~250
GIV
~50
tubulin

Figure S3

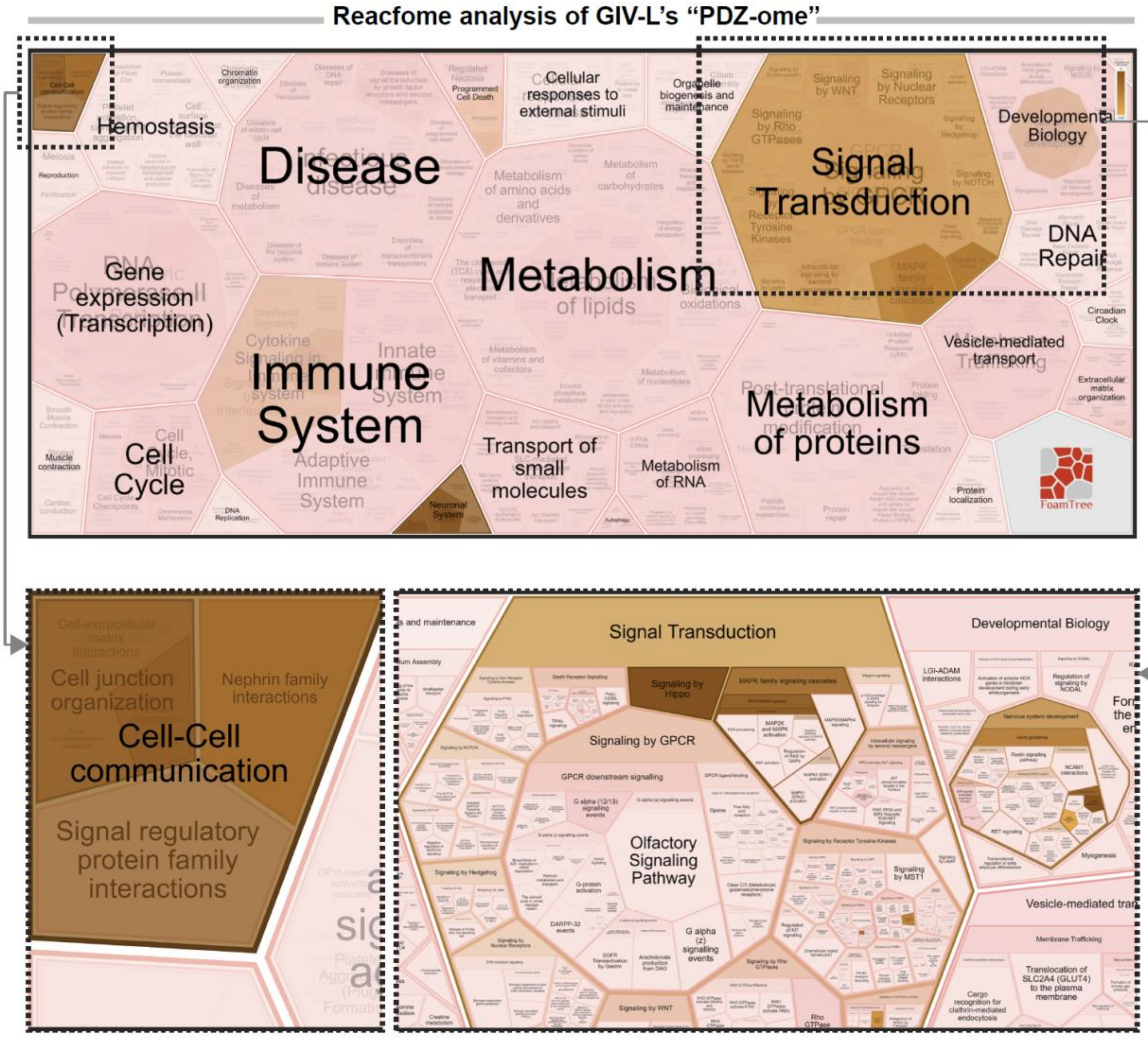

Pathway name	Entities found	Entities Total	Entities ratio	Entities pValue	Entities FDR	Reactions found	Reactions total	Reactions ratio	Species name
Assembly and cell surface presentation of NMDA receptors	5	49	0.003	3.15E-8	4.54E-6	7	23	0.002	Homo sapiens
Ras activation upon Ca2+ influx through NMDA receptor	4	27	0.002	1.95E-7	8.09E-6	2	2	0	Homo sapiens
Negative regulation of NMDA receptor-mediated neuronal transmission	4	27	0.002	1.95E-7	8.09E-6	1	4	0	Homo sapiens
Unblocking of NMDA receptors, glutamate binding and activation	4	28	0.002	2.25E-7	8.09E-6	5	5	0	Homo sapiens
Long-term potentiation	4	31	0.002	3.36E-7	1E-5	3	7	0.001	Homo sapiens
CREB1 phosphorylation through NMDA receptor-mediated activation of RAS signaling	4	39	0.003	8.34E-7	2E-5	2	7	0.001	Homo sapiens
Activation of NMDA receptors and postsynaptic events	5	114	0.008	1.98E-6	4E-5	18	71	0.005	Homo sapiens
Protein-protein interactions at synapses	4	93	0.006	2.52E-5	4.54E-4	13	33	0.003	Homo sapiens
Post NMDA receptor activation events	4	96	0.007	2.85E-5	4.56E-4	5	39	0.003	Homo sapiens
Neurotransmitter receptors and postsynaptic signal transmission	5	232	0.016	6E-5	8.35E-4	21	109	0.008	Homo sapiens
NrCAM interactions	2	7	0	8E-5	1.03E-3	1	4	0	Homo sapiens
RUNX1 regulates expression of components of tight junctions	2	8	0.001	1.03E-4	1.24E-3	2	6	0	Homo sapiens
Neurexins and neuroligins	3	60	0.004	1.86E-4	1.95E-3	12	19	0.001	Homo sapiens
Apoptotic cleavage of cell adhesion proteins	2	11	0.001	1.95E-4	1.95E-3	2	10	0.001	Homo sapiens
Activation of Ca-permeable Kainate Receptor	2	13	0.001	2.71E-4	2.44E-3	2	2	0	Homo sapiens
Ionotropic activity of kainate receptors	2	14	0.001	3.15E-4	2.83E-3	2	4	0	Homo sapiens
Transmission across Chemical Synapses	5	352	0.024	4.13E-4	3.3E-3	23	162	0.012	Homo sapiens
Signaling by Hippo	2	22	0.001	7.7E-4	5.88E-3	4	30	0.002	Homo sapiens
Synaptic adhesion-like molecules	2	23	0.002	8.4E-4	5.88E-3	1	8	0.001	Homo sapiens
L1CAM interactions	3	130	0.009	1.73E-3	1.09E-2	2	54	0.004	Homo sapiens

Figure S4

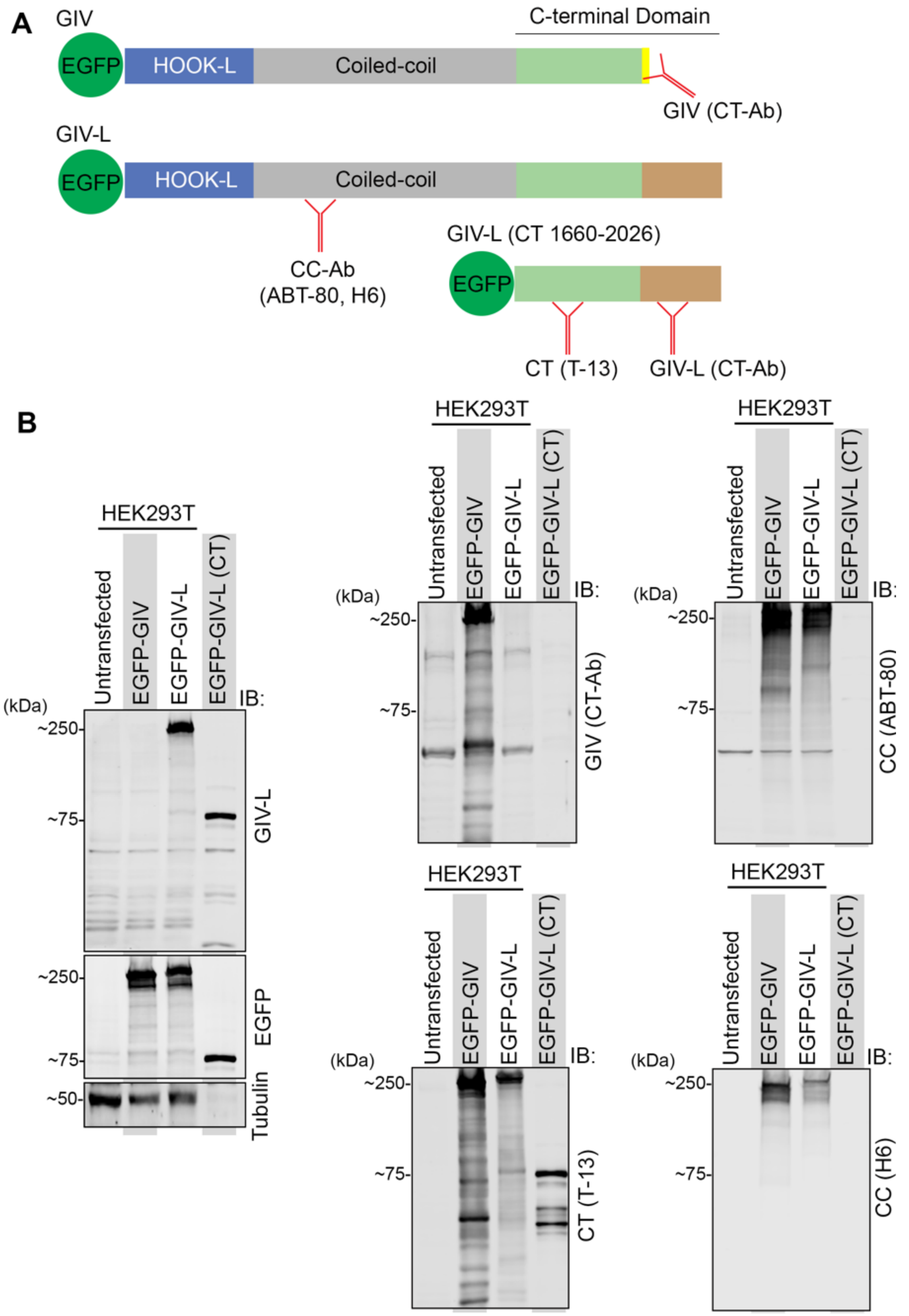
A
GIV
C-terminal Domain
EGFP
HOOK-L
Coiled-coil
GIV (CT-Ab)
GIV-L
EGFP
HOOK-L
Coiled-coil
CC-Ab
(ABT-80, H6)
GIV-L (CT 1660-2026)
EGFP
CT (T-13)
GIV-L (CT-Ab)
B
HEK293T
Untransfected
EGFP-GIV
EGFP-GIV-L
EGFP-GIV-L (CT)
IB:
(kDa)
~250-
~75-
~50-
GIV-L
EGFP
Tubulin
GIV (CT-Ab)
CC (ABT-80)
CT (T-13)
CC (H6)

Key Resource Table

REAGENT or RESOURCE	SOURCE	IDENTIFIER
**Antibodies**		
Rabbit polyclonal anti-Gαi3 (C-10)	Santa Cruz Biotechnology	N/A
Rabbit polyclonal anti-ZO1	GeneTex	GTX108613
Rabbit polyclonal anti-ZO1	GeneTex	GTX108627
Rabbit polyclonal anti-PARD3	Proteintech	11085-1-AP
Rabbit polyclonal anti-E-cadheren	Santa Cruz Biotechnology	sc-7870
Rabbit polyclonal anti-β-tubulin	Santa Cruz Biotechnology	sc-9104
Rabbit polyclonal anti-GIV (Girdin) (T-13)	Santa Cruz Biotechnology	sc-133371
Rabbit polyclonal anti-GIV (Girdin) (CC-Ab)	Millipore Sigma	ABT80
Rabbit polyclonal anti-GIV (CT-Ab)	*This paper*	N/A
Rabbit polyclonal anti-GIV-L (CT-Ab)	*This paper*	N/A
Mouse monoclonal anti-GIV (Girdin) (H-6)	Santa Cruz Biotechnology	sc-393757
Mouse monoclonal anti-β-catenin	Santa Cruz Biotechnology	sc-7963
Mouse monoclonal anti-GAPDH	Santa Cruz Biotechnology	sc-365062
Mouse monoclonal anti-αE-catenin	Santa Cruz Biotechnology	sc-9988
Mouse monoclonal anti-FLAG	Millipore Sigma	MAB3118
Mouse monoclonal anti-FLAG (hybridoma)	Purified in house	N/A
Mouse monoclonal anti-GST	GenScript	A00865
Mouse monoclonal anti-Myc	Cell Signaling Technology	2276S
Mouse monoclonal anti-Myc (hybridoma)	Purified in house	N/A
Mouse monoclonal anti-Dvl	Santa Cruz Biotechnology	sc-166303
Mouse monoclonal anti-α-tubulin	Santa Cruz Biotechnology	sc-5286
Mouse monoclonal anti-EGFP	Santa Cruz Biotechnology	Sc-9996
Goat anti-Rabbit IgG (680)	LI-COR Biosciences	926-68071
Goat anti-Rabbit IgG, Alexa Fluor 594 conjugated	ThermoFisher Scientific	A11072
Goat anti-Mouse IgG (800)	LI-COR Biosciences	926-32210
Goat anti-Mouse IgG, Alexa Fluor 488 conjugated	ThermoFisher Scientific	A11017
ImmPRESS® HRP Horse Anti-Rabbit IgG Polymer Detection Kit, Peroxidase	Vector Laboratories	MP-7401
Anti-Digoxigenin-AP	Roche	11093274910
**Biological Samples and Cell Lines**		
DLD1	ATCC	CCL-221
HCT116	ATCC	CCL-247
Caco-2	ATCC	HTB-37
HeLa	ATCC	CCL-2
HEK293T	ATCC	CRL-11268
Caco-2 ShGIV	Biehler, et. al., 2020	N/A
Caco-2 ShScrambled	Biehler, et. al., 2020	N/A
**Chemicals, Kits, Recombinant Proteins, and Plasmids**		
Streptavidin, Alexa Fluor® 680 conjugate	ThermoFisher Scientific	S21378
Streptavidin, Alexa Fluor® 594 conjugate	ThermoFisher Scientific	S11227
Streptavidin Magnetic Beads	ThermoFisher Scientific	88816
Biotin	Sigma-Aldrich	B4639-500MG

Coelenterazine-h	Promega	S2011
DIG RNA Labeling Mix	Roche	11277073910
T7 RNA polymerase	Promega	P2075
MTT	Millipore Sigma	475989-1GM
Guava Cell Cycle Reagent	Millipore Sigma	4700-0160
ImmPACT® DAB Substrate, Peroxidase (HRP)	Vector Laboratories	SK-4105
Dead Cell Apoptosis Kit with Annexin V Alexa Fluor™ 488 & Propidium Iodide (PI)	ThermoFisher Scientific	V13241
DAPI (4',6-Diamidino-2-Phenylindole, Dilactate)	Thermo Fisher Scientific	D3571
pSpCas9(BB)-2A-Puro (PX459) V2.0	Addgene	62988
pcsDest2-GST	*This paper*	N/A
pcsDest2-GST GIV-L CT (1660-2026)	*This paper*	N/A
pcsDest2-EGFP	*This paper*	N/A
pcsDest2-EGFP-zGIV-CT	*This paper*	N/A
pcsDest2-EGFP-zDaple-CT	*This paper*	N/A
myc-pcDNA 3.1 (+) – GIV (full length)	*This paper*	N/A
myc-pcDNA 3.1 (+) – GIV-F1685A (full length)	*This paper*	N/A
myc-pcDNA 3.1 (+) – GIV-L (full length)	*This paper*	N/A
myc-pcDNA 3.1 (+) – GIV-L-F1685A (full length)	*This paper*	N/A
myc-pcDNA 3.1 (+) – GIV-L-ΔPBM (full length)	*This paper*	N/A
myc-pcDNA 3.1 (+) – GIV-L- F1685A /ΔPBM (full length)	*This paper*	N/A
pcDNA3.1-N-term FLAG-PARD3 (full length)	Peng Zhang, et. al., 2016	N/A
pcDNA3.1-N-term FLAG-PARD3 (ΔPDZ 1)	Peng Zhang, et. al., 2016	N/A
pcDNA3.1-N-term FLAG-PARD3 (ΔPDZ 2)	Peng Zhang, et. al., 2016	N/A
pcDNA3.1-N-term FLAG-PARD3 (ΔPDZ 3)	Peng Zhang, et. al., 2016	N/A
pcDNA3.1(+)-hGai1(91)-RLuc2	Brown et al. 2010	N/A
mVenus-hGBB1	Brown et al. 2016	N/A
mVenus-hGBG2	Brown et al. 2016	N/A
**Software**		
ImageJ	National Institute of Health	https://imagej.net/Welcome
DAVID 6.8	DAVID Bioinformatics Resources	https://david.ncifcrf.gov/home.jsp
Molsoft ICM v3.8-6	Molsoft LLC	http://www.molsoft.com/index.html

**Table 1.** Search parameters.
Search Engine Name: PEAKS
Parent Mass Error Tolerance: 15.0 ppm
Fragment Mass Error Tolerance: 0.4 Da
Precursor Mass Search Type: monoisotopic
Enzyme: Trypsin
Max Missed Cleavages: 3
Non-specific Cleavage: both
Fixed Modifications:
Carbamidomethylation: 57.02
Variable Modifications:
Acetylation (K): 42.01
Acetylation (Protein N-term): 42.01
Acetylation (N-term): 42.01
Amidation: -0.98
Beta-methylthiolation: 45.99
Biotinylation: 226.08
Carbamylation: 43.01
Carboxymethyl: 58.01
and 304 more...
Max Variable PTM Per Peptide: 3
Database: Human Uniprot (2018) # protein sequences 21059
Taxon: All
Contaminant Database: contaminants
Searched Entry: 7000
FDR Estimation: Enabled
De novo score (ALC%) threshold: 15
Peptide hit threshold (-10logP): 30.0
Peaks run ID: 14
Merge Options: no merge
Precursor Options: corrected
Charge Options: no correction
Filter Options: no filter
Process: true
